# Modeling Contact Inhibition of Locomotion of Colliding Cells Migrating on Micropatterned Substrates

**DOI:** 10.1371/journal.pcbi.1005239

**Published:** 2016-12-16

**Authors:** Dirk Alexander Kulawiak, Brian A. Camley, Wouter-Jan Rappel

**Affiliations:** 1 Institut für Theoretische Physik, Technische Universität Berlin, Berlin, Germany; 2 Department of Physics, University of California, San Diego, San Diego, California, United States of America; Oxford, UNITED KINGDOM

## Abstract

In cancer metastasis, embryonic development, and wound healing, cells can coordinate their motion, leading to collective motility. To characterize these cell-cell interactions, which include contact inhibition of locomotion (CIL), micropatterned substrates are often used to restrict cell migration to linear, quasi-one-dimensional paths. In these assays, collisions between polarized cells occur frequently with only a few possible outcomes, such as cells reversing direction, sticking to one another, or walking past one another. Using a computational phase field model of collective cell motility that includes the mechanics of cell shape and a minimal chemical model for CIL, we are able to reproduce all cases seen in two-cell collisions. A subtle balance between the internal cell polarization, CIL and cell-cell adhesion governs the collision outcome. We identify the parameters that control transitions between the different cases, including cell-cell adhesion, propulsion strength, and the rates of CIL. These parameters suggest hypotheses for why different cell types have different collision behavior and the effect of interventions that modulate collision outcomes. To reproduce the heterogeneity in cell-cell collision outcomes observed experimentally in neural crest cells, we must either carefully tune our parameters or assume that there is significant cell-to-cell variation in key parameters like cell-cell adhesion.

## Author Summary

Many cells cooperate with their neighbors to move as a group. However, the mechanisms of these cell-cell interactions are not well understood. One experimental tool to analyze interactions is to allow cells to collide with one another, and see what happens. In order to better understand what features these experiments measure, we develop a computational model of cell-cell collisions, and identify the biochemical and mechanical parameters that lead to different outcomes of collisions. We can recreate all known types of collisions seen in experiments, including cells reversing on contact, sticking, or walking past each other. Our model suggests that what happens in a collision may depend strongly on the mechanical forces between the two cells.

## Introduction

Collective cell migration, in which cells crawl together in a coordinated way, is important for a wide range of biological functions, including the immune system [1], the early development of embryos [2] and cancer invasion [3]. The cell-cell interactions that drive collective behavior can also create new dynamics not seen in isolated crawling cells. This includes fingering instabilities [4–6], cell clusters that exhibit emergent chemotaxis in the absence of individual cell chemotaxis [7–10] or groups of cells which can be governed by a few leader cells [11]. How this collective behavior emerges is an ongoing and active area of research [12, 13].

One well-studied way in which cells interact with one another to create collective motion is contact inhibition of locomotion (CIL), which was named and characterized by Abercombie more than 50 years ago [14], with observations dating back as far as 1921 [15]. CIL describes the tendency of a cell to change its direction of motion after contact with another cell. CIL can play a central role in the coordination of collective migration of cells, including the cluster-level polarization of neural crest cells *in vivo* [16] and the dispersal of hemocytes in the early stages of Drosophila development [17]. CIL may also play a role in cancer [18].

A powerful and commonly used technique to analyze CIL *in vitro* is the collision assay, where the velocity of pairs of cells before and after collision is measured [16]. However, these assays can have low efficiency, since cell-cell collisions are rare. Recently, several groups have developed 1D collision assays, where cell motion is restricted to an adhesive micropatterned stripe, increasing efficiency and ensuring a reproducible collision geometry [19–22]. These assays, originally used to study cell motility in the presence of confinement [23, 24], can be used to study outcomes of cell-cell collision and to identify critical molecular mediators of CIL [20, 22, 25, 26]. The experiments show that head-on collision of two cells can result in four possible outcomes: [19, 20]:

1. **Reversal** Both cells reverse their polarization after collision, detach, and reverse their migration direction.
2. **Sticking** The cells collide and adhere, resulting in a non-motile pair of cells.
3. **Walk-past** Cells collide, move past each other and continue in their original direction.
4. **Chaining** Upon collision, cells form a pair, collectively migrating along the pattern.

In the case of Xenopus cranial neural crest cells, Scarpa *et al.* were able to analyze a large number of cell-cell collisions and to generate quantitative statistics for the possible outcomes [20]. These experiments reveal that the majority of cell collisions resulted in reversals, a smaller fraction of collisions resulted in sticking, walk-past was uncommon and chaining was not observed (see Table 1). However, chaining-like behavior (cells following one another on contact) was observed *in vivo* in chick cranial neural crest cells [27].

**Table 1:** Basic experimental observations.

Outcome	Experimental Percentage	Simulation Percentage
Reversal	73.3%	69%
Sticking	25.1%	27%
Walk-past	1.6%	4%

In this work, we will use a computational model to evaluate the chemical and mechanical factors that control the interactions of motile, eukaryotic cells exhibiting CIL on narrow micropatterned stripes. Our approach, which extends the phase field technique presented in [28], models the mechanics of a changing cell shape as well as the biochemical polarity of the cell. The primary biochemistry we are interested in is cell polarity—i.e. what determines the cell “front” and thus the direction in which the cell is migrating. To do this, we use a minimal model of Rho GTPase kinetics [29] to describe the dynamics of a polarity protein, which we will assume is Rac, which is activated at the front of the cell. CIL is incorporated through the assumption that cell-cell contact leads to local generation or activation of a Rac inhibitor. We will simulate a large number of cell-cell collisions on narrow micropatterned stripes. Our aim is to probe which properties in the cell-cell interactions are responsible for the different outcomes observed in the experiments.

## Model

We extend our earlier modeling work [28, 30], describing cells as two-dimensional objects confined by an interface with a perimeter-dependent line tension *γ* and a bending modulus *κ*. We model two chemical species, *ρ*(**r**, *t*) and *I*(**r**, *t*) inside the cell. *ρ*(**r**, *t*) is the density of membrane-bound (activated) Rac, which leads to actin polymerization. Rac therefore determines the biochemical polarity: the front (back) of the cell is specified by a high (low) value of *ρ*(**r**, *t*). The fluctuating inhibitor *I*(**r**, *t*) controls the persistence of motion of the cell. Both chemical species are described with reaction-diffusion equations within the cell. This section outlines the elements of the model; numerical and implementation details are available in the Supplemental Information (S1 Text).

### Cell mechanics

The interface of a cell *i* is tracked by an auxiliary phase field *ϕ*^(*i*)^(**r**, *t*), which varies smoothly between *ϕ* = 0 (outside of the cell) and *ϕ* = 1 (inside) over a length scale *ϵ*; the cell interface is given by the contour *ϕ* = 1/2. Assuming any fluid flow can be neglected and that the interface is only driven by local forces, the motion of the cell interface is given by

$$\partial_{t}\phi^{\left(i\right)}\left(r,t\right)=\frac{1}{\tau }\left(\alpha \rho (r,t)\chi (r)-\beta \right)\left|\nabla \phi^{\left(i\right)}\right|-\frac{1}{\tau \epsilon }\frac{\delta H}{\delta \phi^{\left(i\right)}}$$ (1)

from a force balance argument [28, 30]. Here, *τ* is a friction coefficient. A full set of parameters and their values is given in S1 Table. We note that many groups have recently modeled both single [30–36] and collective [28, 37–39] cell motility with phase fields.

The first term on the right hand side of Eq 1 describes the active motion of the cell, arising from forces caused by actin polymerization at the leading edge and myosin-driven contraction of the cytoskeleton at the cell rear [40]. This arises because the first term of Eq 1 pushes the cell front outward where *ρ* is large (*αρ* > *β*) and contracts at the rear where *ρ* is low (*β* > *αρ*). To model the effect of the adhesive micropattern, we assume that cells only create protrusion if they are able to adhere to the underlaying substrate. This is implemented by including *χ*(**r**), which takes on values between *χ*(**r**) = 0 (cell cannot adhere) and *χ*(**r**) = 1 (cell can fully adhere). A more in-depth motivation can be found in [41].

In absence of the active motion term in Eq 1, the phase field *ϕ* will minimize a Hamiltonian *H* = *H*_*single*_ + *H*_*cell*−*cell*_. The single cell Hamiltonian is

$$H_{single}=\gamma \left(P\right)\int d^{2}r\left[\frac{\epsilon }{2}{\left|\nabla \phi \right|}^{2}+\frac{G(\phi )}{\epsilon }\right]+\int d^{2}r\frac{\kappa }{2\epsilon }{\left[\epsilon \nabla^{2}\phi -\frac{G^{\prime }\left(\phi \right)}{\epsilon }\right]}^{2},$$ (2)

where *γ*(*P*) is the interface tension and *κ* the bending modulus. The double-well potential *G*(*ϕ*) = 18*ϕ*^2^(1 − *ϕ*)^2^ stabilizes the two phases *ϕ* = 0 (outside of the cell) and *ϕ* = 1 (inside). In the sharp interface limit *ϵ* → 0 and with a perimeter-independent interface tension, it is known that *H*_*single*_ is equivalent to the Canham-Helfrich Hamiltonian [42, 43] (see discussion in [28, 31]). *γ*(*P*) depends on the cell perimeter *P* and has the form

$$\gamma \left(P\right)=\gamma_{0}\cdot \left\{\begin{array}{cc} 1+\gamma_{per}\times \left(P-P_{cr}\right), & \text{if}\;P\geq P_{cr}\\ 1, & \text{otherwise}. \end{array}\right.$$

The perimeter is calculated as *P* = ∫*d*^2^ *r*|∇*ϕ*|. Here, *P*_*cr*_ is a critical perimeter, and for perimeter values above this parameter cells have a component to their perimeter energy that behaves as an elastic membrane with an associated elastic energy *H*_*el*_ ∼ (*P* − *P*_*cr*_)^2^. For cell perimeters below *P*_*cr*_ the line tension is constant as is appropriate for a fluid membrane [42, 44]. One reason we have added this aspect to our model is that when the cell-cell adhesion is very strong it can overcome interface tension, leading to a situation where it is energetically favorable for a pair of cells to increase their perimeter without limitation. Throughout this work we use *γ*_*per*_ = 0.5*μm*^−1^ and *P*_*cr*_ = 58*μm*. Here, *P*_*cr*_ is slightly larger than the unperturbed perimeter of a moving single cell, which is *P* ≈ 56.5*μm* for our default parameters. Note that if *P* increases two microns above *P*_*cr*_, *γ*(*P*) doubles. We have not found any qualitative changes in the collision outcomes for different values of these parameters, as long as the growth of *P* without limitation is prevented. However, we did not conduct systematic variations of these parameters.

The cell-cell interaction part of the Hamiltonian includes two physical interactions, volume exclusion and cell-cell adhesion:

$$H_{cell-cell}=\sum_{i\neq j}\int d^{2}r\left[\frac{g}{2}\phi^{\left(i\right)}\left(r,t\right)\phi^{\left(j\right)}\left(r,t\right)-\frac{\sigma \epsilon^{3}}{4}|\nabla \phi^{\left(i\right)}{|}^{2}{\left|\nabla \phi^{\left(j\right)}\right|}^{2}\right].$$ (3)

The first term excludes volume by penalizing overlap between different cells with strength *g*. The second term, which models adhesion, favors contact between the membranes of different cells. The strength of this interaction is set by *σ*. We note that as a consequence of our phase field description of the cells, altering the strength of repulsion *g* or adhesion *σ* can also change the structure of the interface where cells overlap, i.e. how sharply the interface transitions from *ϕ* = 0 to *ϕ* = 1. This effect would not appear in a sharp-interface model.

### Single cell biochemistry

The chemical concentrations within the cell are modeled with reaction-diffusion equations of the type:

$$\partial_{t}u\left(r,t\right)=D_{u}\nabla^{2}u\left(r,t\right)+f_{u}\left(u\left(r,t\right)\right).$$ (4)

As shown in [45, 46], these equations can be solved in a complex geometry characterized by a phase field *ϕ*. They then have the form

$$\partial_{t}\left(\phi \left(r,t\right)u\left(r,t\right)\right)=\nabla \left[\phi D_{u}\nabla u\right]+\phi f_{u}\left(u\right).$$ (5)

In the sharp interface limit, Eq 5 is equivalent to Eq 4 with Neumann (no-flux) boundary conditions.

Accordingly, our equations for the membrane-bound state *ρ*(**r**, *t*) and the inhibitor *I*(**r**, *t*) in each cell *i* are

$$\begin{array}{c} \partial_{t}\left(\phi^{\left(i\right)}\rho^{\left(i\right)}\right)=\nabla \left[\phi^{\left(i\right)}D_{\rho }\nabla \rho^{\left(i\right)}\right]+\phi^{\left(i\right)}f_{\rho }\left(\rho^{\left(i\right)},\rho_{cyt}^{\left(i\right)},I^{\left(i\right)}\right),\\ \partial_{t}\left(\phi^{\left(i\right)}I^{\left(i\right)}\right)=\nabla \left[\phi^{\left(i\right)}D_{I}\nabla I^{\left(i\right)}\right]+\phi^{\left(i\right)}f_{I}\left(I^{\left(i\right)},\left\{\phi \right\},\left\{\rho \right\}\right), \end{array}$$ (6)

with *D*_*ρ*,*I*_ being diffusion coefficients and *f*_*ρ*,*I*_ reaction terms.

*f*_*ρ*_ describes the exchange between the active membrane bound state *ρ*(**r**, *t*) and an inactive uniform cytosol pool *ρ*_*cyt*_(*t*) of the Rho GTPase Rac, in a modification of the wave-pinning scheme developed by Mori et al. [29]:

$$f_{\rho }\left(\rho^{\left(i\right)},\rho_{cyt}^{\left(i\right)},I^{\left(i\right)}\right)=k_{b}\left(\frac{{\left(\rho^{\left(i\right)}\right)}^{2}}{K_{a}^{2}+{\left(\rho^{\left(i\right)}\right)}^{2}}+k_{a}\right)\rho_{cyt}^{\left(i\right)}-k_{c}\left[1+\frac{I^{\left(i\right)}\left(r,t\right)}{I_{0}}\right]\rho^{\left(i\right)}$$ (7)

$$\rho_{cyt}^{\left(i\right)}=\frac{N_{tot}-\int d^{2}r\phi^{\left(i\right)}\rho^{\left(i\right)}}{\int d^{2}r\phi^{\left(i\right)}}$$ (8)

with *I*_0_ = 1*μm*^−2^.

The rate in Eq 7 models three basic processes: 1) cytosolic *ρ* binding to the membrane at base rate *k*_*a*_ *k*_*b*_, 2) cooperative recruitment of *ρ* from the cytosol to the membrane, and 3) *ρ* detachment from the membrane to the cytosol with the rate *k*_*c*_(1 + *I*(**r**, *t*)/*I*_0_); the inhibitor *I*(**r**) specifies the difference in the base detachment rate *k*_*c*_. These processes conserve the total number of Rac molecules *N*_*tot*_ = ∫*d*^2^ *r*(*ρ*(**r**) + *ρ*_*cyt*_)*ϕ*(**r**). Using this conservation, we find Eq 8 by assuming that the cytosolic state diffuses quickly on the time scales we study, and treating it as spatially constant.

The reaction term for the inhibitor, *f*_*I*_, is written as:

$$f_{I}\left(I^{\left(i\right)},\left\{\phi \right\},\left\{\rho \right\}\right)=-k_{-I}I^{\left(i\right)}\left(r,t\right)+f_{I}{\left(\left\{\rho \right\},\left\{\phi \right\}\right)}_{cell-cell}+\eta \xi^{\left(i\right)}\left(r,t\right)$$ (9)

$$\langle \xi \left(r,t\right)\xi \left(r^{\prime },t^{\prime }\right)\rangle =\eta^{2}\delta^{2}\left(r-r^{\prime }\right)\delta \left(t-t^{\prime }\right).$$ (10)

Here, the first term describes inhibitor decay with rate *k*_−*I*_ and the second term models CIL through inhibitor generation by cell-cell contact (discussed extensively in the next section). The third term describes inhibitor generation by a fluctuating Gaussian Langevin noise *ξ*(**r**, *t*) arising from the intrinsic stochasticity of complex biochemical processes in the cell when there are small numbers of molecules involved [47, 48]. We note that *I*(**r**, *t*) can be negative. Since |*I*|/*I*_0_ is small, positive (negative) *I* corresponds to a small increase (decrease) in the base decay rate of *k*_*c*_ in Eq 7; the effective decay rate never becomes negative. While having a negative concentration appears to be unphysical, this is equivalent to writing an equation for *Y* ≡ *I* + *I*_0_, which is always positive. This can be seen by looking at the sharp-interface equation for the reaction-diffusion model, ∂_*t*_*I* = *D*_*I*_∇^2^ *I* − *k*_−*I*_ *I* + ⋯, which can be transformed into ∂_*t*_*Y* = *D*_*I*_∇^2^ *Y* − *k*_−*I*_(*Y* − *I*_0_) + ⋯, i.e. a reaction-diffusion equation with decay and a basal rate. We have written our model in terms of *I* to show more easily the change from *k*_*c*_, which can be very significant, even if the percentage change is small.

**Fig 1:**
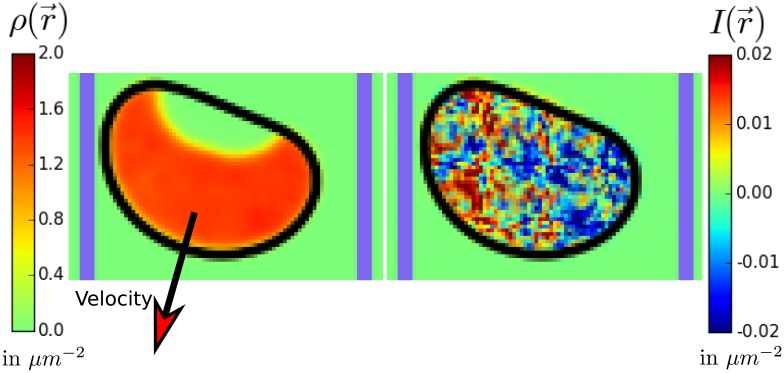
Elements of our model. The cell shape is tracked by a phase field *ϕ*(**r**). The cell boundary (*ϕ* = 0.5 contour line) is plotted in black. On the left side the Rac concentration *ρ*(**r**) is shown, which defines the cell front. The inhibitor level *I*(**r**) is plotted on the right. To limit the internal fields to the inside of the cell, we plot *I*(**r**) × *ϕ*(**r**) (*ρ*(**r**) × *ϕ*(**r**), respectively). Throughout this work we use the shown color scales. To indicate the (static) micropattern, the contour line with *χ*(**r**) = 0.5 is displayed as a thick blue line.

The dynamics of these reaction-diffusion equations lead to a stable profile with a high value of *ρ*(**r**, *t*) defining the front of the cell and a low value indicating the back. The inhibitor’s effect on *ρ* will generally reorient the front of the cell away from high *I*. The resulting distribution of *ρ*(**r**) and *I*(**r**) can be seen in Fig 1 where we show a cell moving down a 1D stripe. The amplitude of the stochastic noise *η* controls the persistence of a single cell’s crawling motion [28]. For *η* = 0 the cell will crawl persistently in one direction and with increased levels of *η* the movement is more erratic.

### Biochemistry of cell-cell interactions

**Fig 2:**
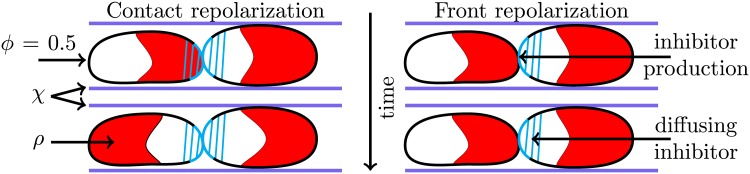
Sketch of the impact of CR (left) and FR (right) repolarization mechanisms on a head-tail collision. Contour lines of *ϕ* are shown in black. The front of the cells with a high value of *ρ* is marked with red. The solid blue line shows edges of the micropattern *χ*. Cyan parts of the cell boundary mark inhibitor production near the cell edge from CR/FR. The inhibitor diffuses to other parts of the cell (cyan stripes) and inhibits *ρ* there. With CR the cells produce inhibitor when they are in contact with any part of another cell. This causes the left cell to repolarize. For FR, there is only inhibitor production when the cell is in contact with the front of another cell. Thus, no cell will repolarize in this head-tail collision.

As mentioned above, and suggested by experimental observations [25], we implement CIL by assuming that additional inhibitor is produced where the phase fields of the two cells overlap. The production of additional inhibitor, coupled with diffusion in the cell interior, can result in cell repolarization away from contact areas [28]. As in earlier computational work [28] and based on experiments using NRK-52E cells [19] we model two distinct mechanisms for CIL, contact repolarization (CR) and front repolarization (FR), and vary their relative importance by the constants *k*_*CR*_ and *k*_*FR*_, respectively. We sketch these mechanisms in Fig 2. Mathematically, the two mechanisms are described by including the following reaction terms for the *i*-th cell:

$$f_{I}^{i}{\left(\left\{\rho \right\},\left\{\phi \right\}\right)}_{cell-cell}=\underset{\text{CR}}{\underset{︸}{k_{CR}S\left(\sum_{j\neq i}^{N_{cell}}\phi^{\left(j\right)}\right)}}+\underset{\text{FR}}{\underset{︸}{k_{FR}S\left(\sum_{j\neq i}^{N_{cell}}\frac{\phi^{\left(j\right)}\rho^{\left(j\right)}-O_{crit}}{\rho^{char}}\right)}},$$ (11)

where *S*(*x*) is a sigmoidal function, *S*(*x*) = max(0, tanh[*x*/*x*_0_]) with *x*_0_ = 0.1 and *N*_*cell*_ the total number of cells.

The first term of Eq 11 describes CR. The production of *I* will start as soon as there is cell-cell overlap and will cease when this overlap becomes negligible. The second term incorporates FR and, contrary to CR, also depends on the polarization of the neighboring cells: it only leads to additional inhibitor production if the *i*-th cell is in contact with another cell and if that other cell has elevated levels of Rac at the point of cell-cell overlap.

We consider the possibility that the contact repolarization and front repolarization interactions occur by different signaling pathways, which set in at different times, e.g. if detection of cell-cell contact and cell front contact occur through different mechanisms. For instance, we could envision that cell-cell contact leads to mechanical force, leading to generation of an inhibitor at contact (i.e. CR), but that FR requires ligand-receptor binding to specifically recognize the front of another cell. These could have different rates, force requirements, or other kinetic details. We treat this possibility generically by allowing the rates *k*_*FR*_ and *k*_*CR*_ to differ, as well as introducing a critical overlap *O*_*crit*_ in Eq 11, which sets a threshold that has to be exceeded for the FR to set in. Unless the overlap between the cells exceeds this threshold, the FR will not generate any inhibitor. When cells approach one another the values of *ϕρ* in the contact region are small and below this threshold. Thus, one of the effects of the critical overlap is that the FR sets in later, when the cells overlap sufficiently and not upon first contact. Therefore, the mechanical interactions between the cells are stronger when the FR sets in. If *O*_*crit*_ is too large the threshold will not be exceeded and the FR does not produce any inhibitor at all. At lower values of *O*_*crit*_, FR only produces inhibitor in regions where the cells overlap reasonably and *ϕρ* of the other cell is sufficiently high. Hence, the distribution of the inhibitor production is different and will be concentrated to these regions. Additionally, as only the overlap which exceeds the threshold yields inhibitor production, the critical overlap will reduce the total amount of produced inhibitor for a given FR strength *k*_*FR*_. Typical values of *ρ*(**r**) at the cell front are around 1.4*μm*^−2^. This is much larger than the value at the back, which is typically around 0.01*μm*^−2^. Usually we choose values of *O*_*crit*_ between 0 and 0.2*μm*^−2^. This ensures that *ρϕ* crosses the threshold and inhibitor is generated when cells are in full front-front contact (i.e. when the contours *ϕ*^(1)^ = 1/2 and *ϕ*^(2)^ = 1/2 are close), but delays the inhibitor production when they are approaching.

We emphasize that these two biochemical interactions have essential differences. For CR, cells only cease the production of inhibitor when they are not in contact anymore. In contrast, with FR a cell stops producing inhibitor when the other cells starts to repolarize. If *O*_*crit*_ > 0, the two interactions will also start their onset at different times in the cell-cell contact.

We note that, for simplicity, we have only included a critical overlap *O*_*crit*_ in the FR interaction. Additional behaviors could potentially be found by including a similar parameter for the CR interaction. We have made this assumption both to reduce the parameter space slightly, and because we view the contact repulsion as a more fundamental effect: detecting cell-cell contact may be much simpler than detecting whether contact is specific to the front of the cell.

## Results

### Model reproduces experimentally observed cell-cell collision types

**Fig 3:**
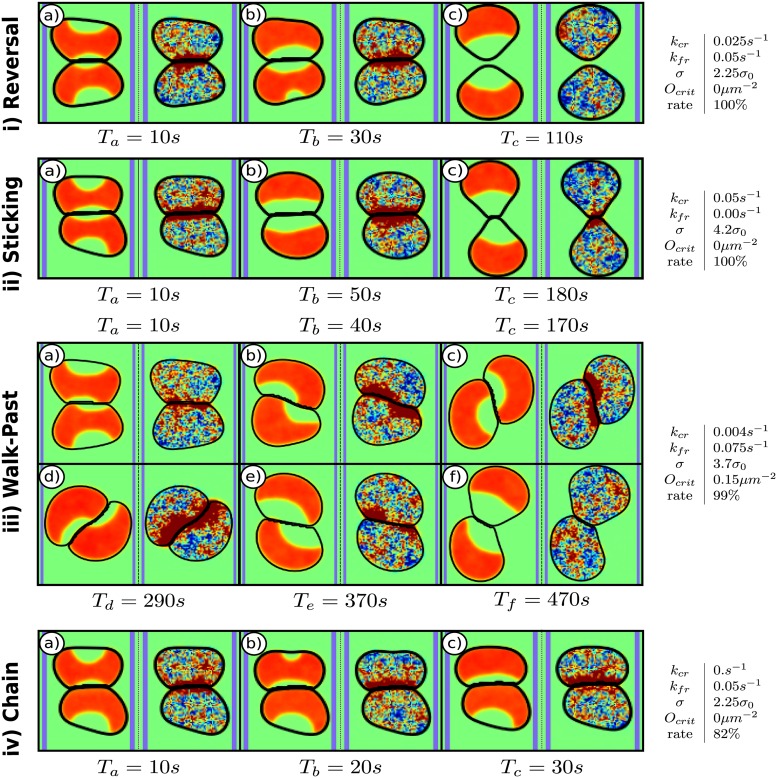
Snapshots of different outcomes. In each panel *ρ*(**r**) is on the left, *I*(**r**) on the right and the edges of the adhesive micropattern are indicated in blue. *α* = 0.4*α*_0_ for all cases. The outcomes are i) reversals, ii) sticking, iii) walk-past, and iv) chaining. Next to each outcome are the parameters of the snapshots and the rate of the outcome for the given parameters based on 100 simulations. We chose the parameters that yield the maximum rate for each outcome. *α*_0_ = 1*pN*/*μm*^3^ and *σ*_0_ = 1*pN*/*μm*. Times are measured relative to the time of first contact.

Our simulations can reproduce all experimental observed cell-cell collision cases (reversal, sticking, walk past, and chaining) by varying only four parameters: adhesion strength *σ*, critical overlap *O*_*crit*_, and the CR and FR strengths *k*_*CR*_ and *k*_*FR*_. In Fig 3, we present representative snapshots of all four cases along with the values of the four key parameters.

1. **Reversal** Fig 3i show a typical cell reversal: upon contact, the cells start to produce inhibitor (Fig 3ia) which leads to a repolarization of both cells (Fig 3ib). When the repolarization is complete, both cells will migrate in opposite directions, away from the site of contact (Fig 3ic). This reversal is shown in S1 Movie. The speed of cells during collisions is shown in S1 Fig and shows a qualitative agreement with the data of [20], with a drop during repolarization and a sharp rise immediately following cell-cell separation followed by a return to pre-collision speeds.
2. **Sticking** Increasing the adhesion can result in cells that stick to each other. In this case, cells repolarize after they collide (Fig 3iia and 3iib) but cannot separate from each other and the cells remain connected and non-migratory (Fig 3iic). (See S2 Movie)
3. **Walk-past** With a fine tuned combination of FR and CR cells can walk past each other. This is shown in Fig 3iii where the strong FR creates an asymmetric repolarization (Fig 3iiia and 3iiib). This asymmetry is amplified and results in cells that retract at one side of the stripe, push forward at the other one (Fig 3iiib) and squeeze past each other (Fig 3iiic and 3iiid). After passing, the cell fronts point in opposite directions (Fig 3iiie) and they separate (Fig 3iiif). (See S3 Movie)
4. **Chaining** For a medium FR and a weak CR cells can form chains, as shown in Fig 3iv. Small asymmetries due to the fluctuating inhibitor and any asymmetries in the collision can lead to one cell repolarizing earlier than the other (Fig 3iva). If the repolarization is fast enough and the FR is weak enough, it can be accomplished before the other cell repolarizes, resulting in both cells moving as a chain (See S4 Movie).

### Collision outcomes are controlled by a combination of mechanical and biochemical parameters

To gain a better understanding of the relative importance of the critical parameters we carry out several parameter sweeps. These sweeps are performed by varying one or two parameters, while keeping the others fixed to their default values (which are given in S1 Table), or otherwise as noted.

#### Proportion of propulsion to adhesion controls transitions from reversal to sticking

**Fig 4:**
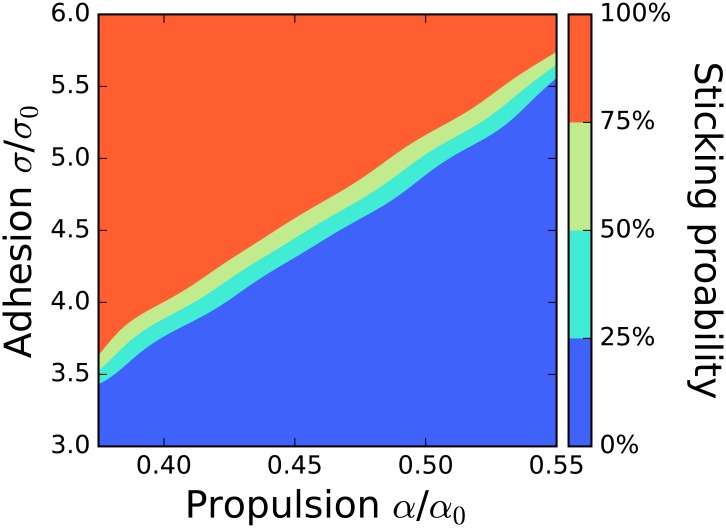
Transition between sticking and reversal is sharp and depends on balance of adhesion and propulsion. We show the fraction of sticking events; all other events are reversals. The parameters are *k*_*CR*_ = 0.1*s*^−1^, *k*_*FR*_ = 0*s*^−1^ and *O*_*crit*_ = 0*μm*^−2^. The simulations run for *T* = 2500*s*. For *α* the step size is 0.025*α*_0_ and for *σ* it is 0.02*σ*_0_ close to the transition and 0.25 further away. We did 100 simulations for points near the transition and otherwise 10.

In a first sweep, we analyze the relative importance of adhesion and propulsion. For this, we vary the cell’s propulsion (*α*) and adhesion strength (*σ*) while keeping *k*_*CR*_ and *k*_*FR*_ fixed. Fig 4 shows the percentage of sticking events in the *α*-*σ* space; all non-sticking events are reversals in this figure. Not surprisingly, we see that for a fixed propulsion strength there is a critical value of the adhesion strength above which cells stick. The transition between sticking and reversal is very sharp and coexistence of both outcomes is only possible in a narrow range of parameter values.

Increasing the propulsion strength *α* moves the sticking/reversal transition to larger adhesion strengths—cells with stronger adhesion require more force to separate. We find that the critical adhesion *σ*_*trans*_, where the portion of both cases is 50%, can be fitted linearly,

$$\sigma_{trans}/\sigma_{0}=-0.8+11.65\alpha /\alpha_{0}.$$ (12)

We note that this transition is slightly sensitive to the length of the simulations *T*, as cells that have stuck together may separate after a long time. For longer simulations, more cells separate and the reversal-sticking transition happens at slightly larger *σ*. This is consistent with the sticking state near the transition being only metastable, with a diverging lifespan as we move to higher *σ*. However, there is no qualitative change in the outcome of the simulations as *T* is lengthened (S2 Fig).

Though we have simulated sticking with *k*_*FR*_ = 0 in Fig 4, we note that the qualitative picture of the phase diagram does not change for *k*_*FR*_ > 0. However, for non-zero values of *k*_*FR*_ we sometimes observe persistently rotating pairs above the sticking transition. We show an example with *k*_*CR*_ = 0*s*^−1^, *k*_*FR*_ = 0.1*s*^−1^ and *O*_*crit*_ = 0.15*μm*^−2^ in S5 Movie. We do not explore these rotating pairs further here.

#### Walk-past requires adhesion, a balance of front and contact repolarization, and a critical overlap

We next determine which parameters are critical for walk-past outcomes. We find that this outcome is only possible if we incorporate three crucial elements: strong, but not too strong, adhesion, the delay of front repolarization through imposing a critical overlap (i.e., *O*_*crit*_ ≠ 0), and a dominance of FR over CR. This careful tuning of parameters is consistent with the experimental observation that walk-past is only rarely observed in the experiments of [20] on neural crest cells.

**Fig 5:**
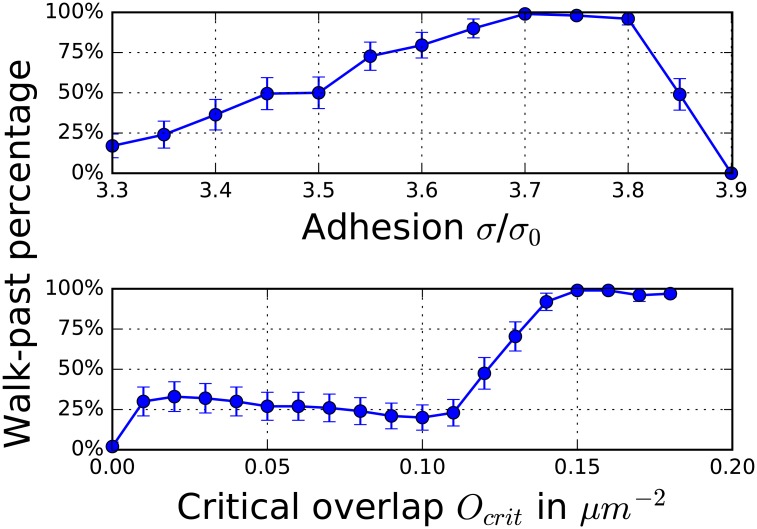
Walk-past depends non-monotonically on adhesion, strongly on critical overlap. Percentage of collisions that lead to walk-past events is shown; 100 simulations are performed for each data point. The error bars are calculated with the binomial proportion confidence interval with the significance level 0.05. The parameters are *O*_*crit*_ = 0.15*μm*^−2^ (upper panel), *σ* = 3.70*σ*_0_ (lower), *α* = 0.4*α*_0_, *k*_*FR*_ = 0.075*s*^−1^ and *k*_*CR*_ = 0.004*s*^−1^.

To quantify the dependence of the walk-past probability on the adhesion strength between cells, the first crucial element, we systematically varied *σ* while keeping all other parameters fixed. For small values of the adhesion strength, most cells show reversals and the walk-past probability is low (Fig 5, upper panel). Increasing the adhesion strength results in increasing probability for walk-past which peaks at *σ* = 3.70*σ*_0_. The walk-past probability stays above 95% until *σ* = 3.80*σ*_0_. Beginning with *σ* = 3.85*σ*_0_ cells can stick together, reducing the percentage of successful walk-past events.

For cells to walk past one another, they must overcome adhesion and separate. Why, then, does increasing adhesion ever increase the rate of walk-past? Walk-past in our model requires that one cell polarizes towards one boundary of the pattern and the other polarizes to the opposite boundary. This asymmetrically “left-right” polarization leads to a coordinated motion that resembles the rotational movement of cell pairs [28, 49]. However, at this stage, cells are also polarized away from contact (Fig 3iiib), and in the absence of adhesion would tend to detach. Stronger adhesion keeps the cells in contact during the exchange, and permits the walk-past to continue. At even higher values of the adhesion parameter, cells will stick and not be able to separate. Notably, even though the contact and front repolarization parameters differ from those in Fig 4, the transition to sticking in Fig 5 (upper panel) occurs at nearly the same adhesion strength, *σ* ≈ 3.86*σ*_0_ when *α* = 0.4*α*_0_.

A second necessary component for the walk-past is to have a front polarization that is delayed. This is accomplished in our model by choosing a value of *O*_*crit*_ larger than zero. In the lower panel of Fig 5 we plot the probability of walk-past as a function of *O*_*crit*_. This probability is negligible for *O*_*crit*_ = 0, increases rapidly for non-zero values of *O*_*crit*_ and saturates at near 100% for *O*_*crit*_ = 0.15*μm*^−2^.

Why does *O*_*crit*_ strongly influence the walk-past rate? The front repolarization mechanism promotes configurations where *ρ* is large in one cell, but small at the contacting point in the other cell. This can create two types of asymmetries: up-down as in chaining (Fig 3ivb) and left-right as in walk-past (Fig 3iiib). A nonzero *O*_*crit*_ has several effects. First, nonzero *O*_*crit*_ introduces a delay before the front repolarization effect occurs. This ensures that cells have a larger common interface before a repolarization sets in. Secondly, as *O*_*crit*_ increases, the sensitivity of FR to changes in *ρ* rises (Eq 11). In addition, the decreased total production of inhibitor due to nonzero *O*_*crit*_ yields a slower repolarization. Together, these effects favor left-right asymmetries and boost the walk-past rate.

**Fig 6:**
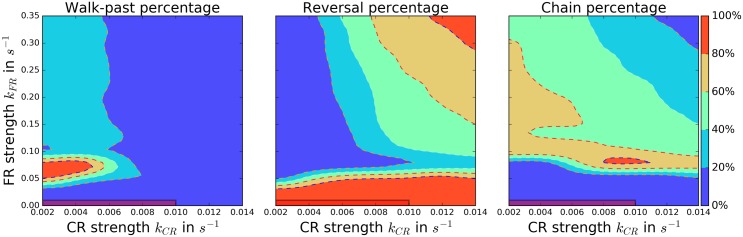
Robust walk-past requires a balance of contact repolarization and front repolarization. Percentage of walk-past (left), reversal (middle), and chaining (right) events. It should be noted that *k*_*FR*_ is an order of magnitude larger than *k*_*CR*_. We don’t include *k*_*CR*_ = 0*s*^−1^ in this figure because these cases can have ambiguous outcomes. In the marked region in the lower left corner mechanical interactions can dominate and the collision outcomes do not always resemble any of the four experimental cases (details for both in text). The parameters are *σ* = 3.70*σ*_0_, *O*_*crit*_ = 0.15*μm*^−2^ and *α* = 0.4*α*_0_. We performed 100 simulations for each point of the grid, which has a step size of 0.002*s*^−1^ for *k*_*CR*_ and 0.025*s*^−1^ for *k*_*FR*_; the color maps have been interpolated.

To determine how the final crucial ingredient (larger FR than CR) affects the walk-past probability we study the *k*_*CR*_-*k*_*FR*_ phase-diagram. Here, however, we choose values for *O*_*crit*_ and *σ* for which the walk-past probabilities in Fig 5 are maximal (*O*_*crit*_ = 0.15*μm*^−2^ and *σ* = 3.70). We find a small region in phase space for which the walk-past probability is significantly larger than 50% (Fig 6, left). For completeness, we also show in Fig 6 the probabilities for both reversals and chaining.

Fig 6 reveals that careful tuning of contact repolarization and front repolarization is necessary to see significant walk-past rates. For a strong CR the high inhibitor production leads to reversals; even a low value of *k*_*CR*_ = 0.016*s*^−1^ is sufficient to prevent the walk-past from happening. Due to the additional inhibitor produced by the CR, the FR is not able to maintain the spatial asymmetry in *ρ* that is needed for the walk-past case. However, unlike the chained state we will discuss below, walk-past rate is maximized by a nonzero contact repolarization. The role of CR here may be to tend to reorient the cells away from each other after they have passed, promoting separation at the late stage. We should note that we do not show the results of simulations with *k*_*CR*_ = 0*s*^−1^ in Fig 6. For this value of *k*_*CR*_ cells are often not able to separate after passing each other and then can pass each other again, sometimes undergoing multiple walk-pasts. This type of behavior can also be observed for *k*_*CR*_ = 0.002*s*^−1^, but it occurs less often. We count cells that walk past each other two times (double walk-past) as walk-past outcomes (see S1 Text and S2 Table, for details).

The necessity of both CR and FR is consistent with our results in [28] that FR promotes rotational motion, while CR suppresses it. Here, we find that a carefully chosen combination of a strong FR and a weak CR enables both the rotational motion which starts the walk-past, and the eventual counter directional alignment that leads to cell separation. The mixture of these two features can yield a high walk-past rate.

We also note that we observe two different characteristic versions of the walk-past. At lower values of *k*_*FR*_ both cells first migrate as a chain in the same direction until the walk-past movement sets in (S7 Movie). At higher ones the walk-past starts immediately at the first contact. For the optimal value *k*_*FR*_ = 0.075*s*^−1^ both versions happen, yielding a high percentage of walk-past events.

In the absence of CIL cells do not produce any inhibitor on contact and only interact mechanically by cell-cell adhesion and repulsion. Then, many collisions don’t resemble any of the four experimental cases. We do observe some reversal-like outcomes, while others are ambiguous with repeated loss of polarization and spontaneous repolarization. This repolarization after cell-cell collisions can occur even if the explicit generation of *I* at the cell-cell contact is negligible. For instance, if two cells collide but fail to initially repolarize, one cell can turn around solely due to internal fluctuations in *I*. Mechanical deformations that change the cell size or shape can also lead to repolarization (see [29, 41], S6 Movie). For very weak biochemical interactions with values of *k*_*CR*_ ≤ 0.01*s*^−1^ and *k*_*FR*_ ≤ 0.01*s*^−1^ collisions are primarily dominated by these events, and are not controlled by the biochemical cell-cell interaction mechanisms of CR and FR; this ambiguous region is shaded in Fig 6. We emphasize that, even when CIL is absent, we do not observe walk-past at large rates—the cells do not walk past one another without some degree of coordination.

#### Contact repolarization generically promotes reversals; tuning front repolarization creates chains

Finally, we determine parameter values for which cell collisions result in chaining. Chaining has been observed in NRK-52E cells, which also exhibit CIL [19]. Comparable behavior can also be seen in neural crest cells *in vivo* in some circumstances [27]. However it was not observed in the experiments by Scarpa *et al.* with Xenopus cranial neural crest cells [20].

**Fig 7:**
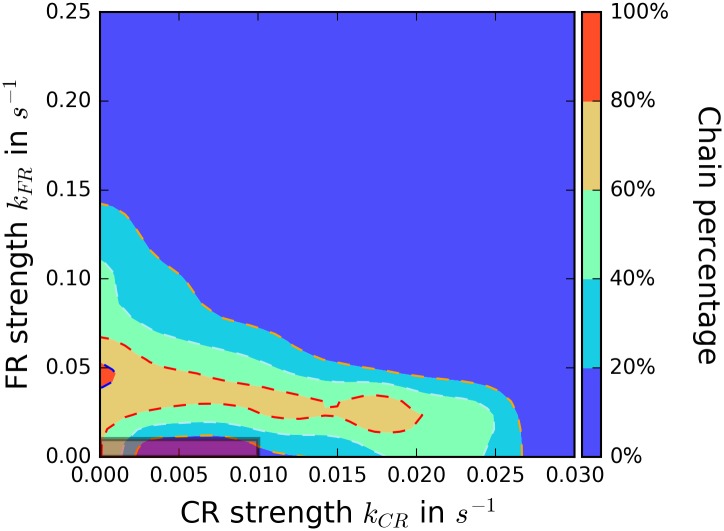
Reversal is robust, but chains require tuning. The percentage of collisions that result in chains is plotted; all other collisions create reversal, except in the marked region with *k*_*CR*_ ≤ 0.01*s*^−1^ and *k*_*FR*_ ≤ 0.01*s*^−1^ where mechanical interactions can dominate (discussed in the text). The parameters are *σ* = 2.25*σ*_0_, *O*_*crit*_ = 0*μm*^−2^ and *α* = 0.4*α*_0_. We did 100 simulations for each point of the grid, which has a step size of 0.0025*s*^−1^ for *k*_*CR*_ and 0.025*s*^−1^ for *k*_*FR*_. It should be noted that *k*_*FR*_ is an order of magnitude larger than *k*_*CR*_.

In Fig 7, we show the percentage of chaining events as a function of *k*_*CR*_ and *k*_*FR*_; non-chaining events in this figure are generally reversals. We see that robust chaining is only possible if contact repolarization is weak, and if front repolarization is at an intermediate strength (note the different scale of the two axes in the phase diagram). This result can be explained by noting that for a pair of cells to form a chain, one cell must repolarize before the other, i.e. the repolarization must be stochastic, as identified by [19]. For large values of *k*_*FR*_, cell repolarization is fast and controlled by the initial front-front contact. Thus, the cells repolarize in opposite directions, resulting in cell reversal. For smaller values of *k*_*FR*_ and small values of *k*_*CR*_, inhibitor production after the collision is reduced. Due to fluctuations, one of the two cells can repolarize before the other resulting in two cells with the same polarization direction. This chain will be stable as long as *k*_*CR*_ is small. Obviously, increasing the value of *k*_*CR*_ will result in rapid and significant inhibitor production when cells collide and, hence, cell reversals.

As in the previous section, mechanical forces rather than biochemical interactions can govern collisions with *k*_*CR*_ ≤ 0.01*s*^−1^ and *k*_*FR*_ ≤ 0.01*s*^−1^ resulting in ambiguous cases that do not always resemble any of the four experimental outcomes.

### Varying the stripe width *d* affects walk-past probability

**Fig 8:**
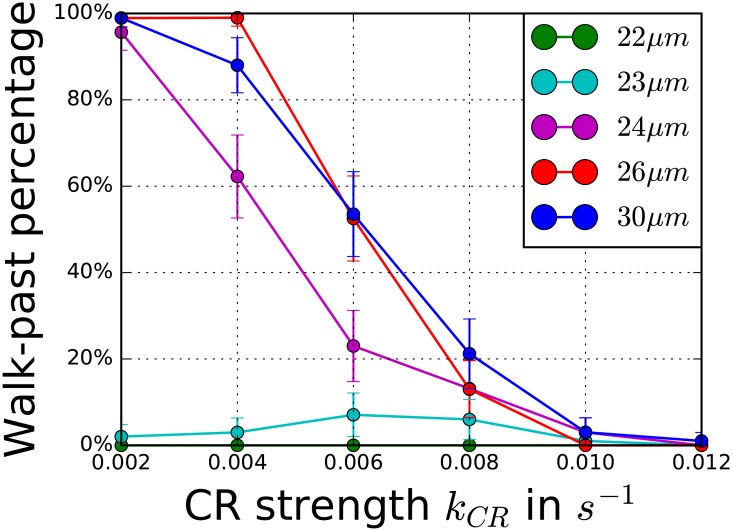
Walk-past percentage may be affected by micropattern width. Percentage of walk-past events for different width *d* of the stripe. We don’t include *k*_*CR*_ = 0*s*^−1^ in this figure because these cases can have ambiguous outcomes (Compare with discussion of Fig 6). We conducted 100 simulations for each data point. The error bars are calculated with the binomial proportion confidence interval with the significance level 0.05. The parameters are *k*_*FR*_ = 0.075*s*^−1^, *σ* = 3.7*σ*_0_, *α* = 0.4*α*_0_ and *O*_*crit*_ = 0.15*μm*^−2^.

Most of the parameters we have varied in our parameter sweeps are not readily accessible in experiments. One experimental parameter that can easily be varied, however, is the width of the adhesive stripe, *d*. Consequently, we tested how narrower and wider stripes change the outcomes of our simulations. Our modeling results predict that varying *d* strongly affects the walk-past transition, as is shown in Fig 8 where we plot the percentage of walk-past events as a function of *k*_*CR*_ for various values of *d*.

Decreasing the stripe width *d* can strongly decrease the walk-past rate. Intuitively, this is clear: if the stripe is too narrow the cells cannot pass each other. Our simulations reveal that this transition is sharp; for example, for *k*_*CR*_ = 0.004*s*^−1^ the maximum walk-past rate drops from ∼60% (*d* = 24*μm*) to ∼4% (*d* = 23*μm*) to 0% (*d* = 22*μm*). Fig 8 also reveals that the dependence of walk-past probability on the stripe width is not necessarily monotonic. For instance, for *k*_*CR*_ = 0.004*s*^−1^ we see that the walk-past probability increases for increasing values of stripe width, reaches a maximum for *d* = 26*μm* and then decreases again. The reason for this decrease in walk-past behavior is the lack of confinement. For larger stripe widths cells sometimes begin the walk-past movement, but repolarize too quickly to complete it, resulting in a reversal instead of a walk-past.

Comparable to Fig 6 cells can walk past each other several times for very small *k*_*CR*_, *k*_*CR*_ = 0.002*s*^−1^. We note that if a cell pair performs two walk-pasts and then separates, we also count this as a walk-past. For more details on the exact classification, please see S1 Text.

### Model can quantitatively reproduce experimental outcome statistics

We next ask the question whether our model can reproduce the quantitative statistics of cell-cell collision found in experiments of neural crest cells which revealed that ∼25% of cell pairs stick, ∼73% of cell pairs reverse, and ∼2% walk past [20]. As is evident in Fig 4, the coexistence region between sticking and reversal requires careful tuning. We manually varied all four relevant parameters and find that we can generate reversal statistics that quantitatively agree with the experiments. Specifically, we find that for *k*_*FR*_ = 0.04*s*^−1^, *k*_*CR*_ = 0.01*s*^−1^, *O*_*crit*_ = 0.15*μm*^−2^, and *σ* = 3.85*σ*_0_ our simulations result in ∼69% reversal, ∼27% sticking, and ∼4% walk-past (see Table 1). Small deviations in these parameter values can cause large changes in outcomes; we will use this to screen for parameters which are critical to the experimental outcomes below.

### Model outcome can be compared to treatment effects

Our model can be used to predict and test the outcome of various biochemical interventions in the experiments. For example, the CIL response in neural crest cells can be blocked with the application of two treatments (DshDEP+ and Y-27632) [16], yielding a reduction in reversal and an increased number of sticking events—an increase to 44% sticking with DshDEP+ and 67% with Y-27632 [20]. In our model, a natural starting assumption for modeling the effect of these treatments in reducing CIL would be decreasing *k*_*CR*_. Can reducing *k*_*CR*_ increase the number of sticking events, or do we need to invoke other effects of these treatments on, e.g. cell-cell adhesion?

To test this hypothesis, we start with the parameter set that produce results consistent with the control experiments of [20] (Table 1) and determine the required change in model parameter values to increase the sticking probability to above 50%, as observed with their intervention experiments. The parameters that are more critical to controlling this outcome are likely candidates for the most relevant effects of treatments that increase sticking. We summarize these results in Table 2 by showing the required change in each parameter values (size and sign). The full results, including the percentage of each outcome as parameters are varied, are shown in S3 Fig.

**Table 2:** Required parameter change to increase the sticking percentage above 50%.

Parameter	Change required for > 50% sticking
*k*_*CR*_	−80%
*k*_*FR*_	+ ∼ 55%
*σ*	+ 0.3%
*O*_*crit*_	-
propulsion strength *α*	− ∼ 0.1%
contraction strength *β*	+ ∼ 1%
line tension *γ*	+ ∼ 1%
Overall activation rate *k*_*b*_	+ ∼ 0.3%
Inhibitor diffusion *D*_*I*_	-

For entries with - we were not able to increase the percentage of sticking cases above 50% by changing that variable within a range of 40%(*D*_*I*_) and 20%(*O*_*crit*_). The changes are relative to the parameter combination which reproduces the experimental outcome: *k*_*FR*_ = 0.04*s*^−1^, *k*_*CR*_ = 0.01*s*^−1^, *O*_*crit*_ = 0.15*μm*^−2^, *σ* = 3.85*σ*_0_, *α* = 0.4*α*_0_, *β* = 0.2*pN*/*μm*, *γ* = 1.8*pN* and *k*_*b*_ = 10*s*^−1^.

Consistent with our hypothesis, we can reproduce the effects of CIL-inhibiting treatments by altering the behavior of the inhibitor generation, either by reducing *k*_*CR*_ or by increasing *k*_*FR*_. For lower values of *k*_*CR*_ we see more sticking and walk-past outcomes and fewer reversals (S3 Fig). This can be understood by realizing that a weaker CR causes the repolarization to be slower, resulting in more cells that stick together. Surprisingly, an increased FR strength yields more sticking outcomes. Why does a stronger biochemical interaction lead to more sticking cases? We can observe two different mechanisms that lead to a pair of sticking cells. A stronger FR results in more cells that start the walk-past movement. The relatively strong CR prevents most of these from completing and the cells repolarize before the walk-past is carried out. These aborted walk-pasts often result in sticking cases. Additionally, more chains form at larger FR. These are unstable and their breakup often yields the sticking outcome. If *k*_*FR*_ is increased further more chains form which become increasingly stable. Hence, the sticking rate decreases again (S3 Fig).

Our simulations also show that other parameters can result in significantly more sticking events. Most of these parameter changes can be explained at an intuitive level. For example, decreasing the propulsion or increasing the contraction strength will make it harder for cells to separate (see Fig 4) and will thus increase the sticking probability. In addition, a stronger line-tension *γ* makes it harder for the cells to push the membrane outwards and will result in more sticking. More surprising, however, is the effect of increasing the activation rate for Rac, *k*_*b*_. Even though a higher activation rate yields a stronger recruitment of *ρ* from the cytosol to the membrane, the maximum level of *ρ* at the cell front does not rise. It is observable that the area of a single, freely migrating cell grows and the velocity drops slightly. The lower velocity even indicates a lower value *ρ* at the cell front and thus a weaker protrusion and more sticking. Taken together, our results suggests that our hypothesis, that CIL-inhibiting treatments strongly regulate the CIL response in [20] through the modification of *k*_*CR*_ and *k*_*FR*_, may need to be extended; we address this further in the Discussion.

### Cell collision phenotypes control collective cell motility on stripes, including cell trains

**Fig 9:**
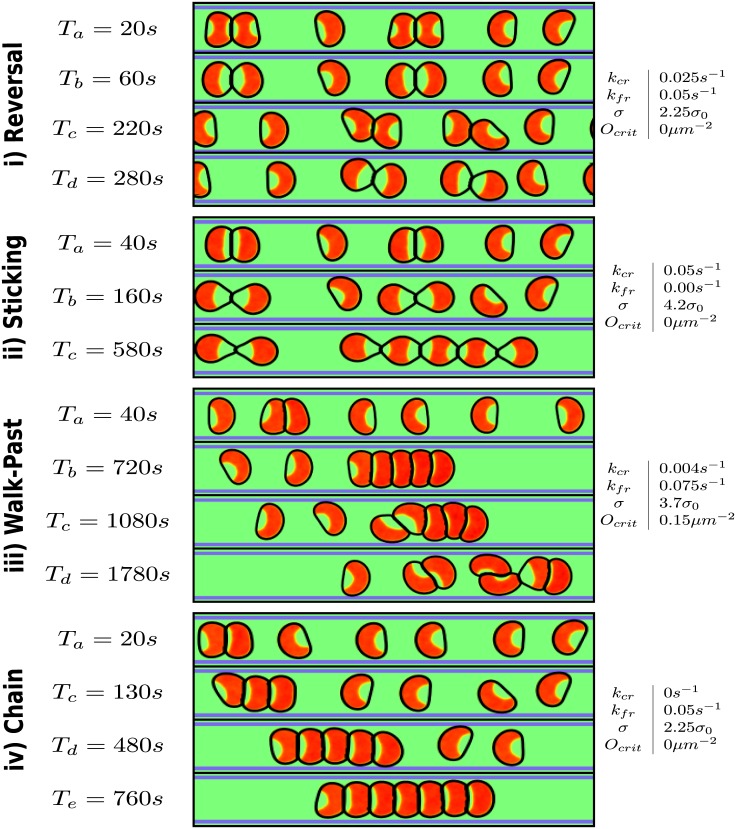
Cell collision phenotype alters collective behavior of cells on stripes. Representative snapshots of multi-cell simulations. Only *ρ*(**r**) is shown. The parameters for i)-iv) correspond to those seen in Fig 3.

Our model is easily extendable to more than two cells to address experimental studies of collective migration on stripes [19]. In Fig 9 we show outcomes of simulations of multiple cells on a single, long stripe for four parameter combinations that each yield a high percentage of one of the possible two-cell collision outcomes. Parameters that promote cell-cell reversal lead to cells that robustly repolarize upon contact with another cell and remain well distributed over the stripe (Fig 9i, S8 Movie).

Parameters that create sticking cell pairs lead to cells that repolarize upon collision and mostly stick with the colliding cell. However, if a single cell collides with a pair of already sticking cells, we can observe that one cell of the pair may detach, resulting in non-motile cells that are mainly in pairs (Fig 9ii, S9 Movie). For larger adhesion strengths, we can also observe larger clusters. For walk-past parameters, cells begin to form trains but instead of having persistent motility as in [19], the trains break up (Fig 9iii, S10 Movie). Finally, for chain parameters, we observe the formation of persistent cell trains (Fig 9iv, S11 Movie). The trains form in a similar manner to the experiment of Ref. [19]: isolated cells collide and form moving chains. Subsequent collision with other isolated cells result in even larger chains.

## Discussion

We have presented a model to describe the crawling and collision dynamics of cells on a micropatterned stripe. We applied the modeling approach from [28], including modeling contact-related signaling events known to occur in neural crest cells. This model can reproduce the four distinct phenotypes of cell-cell collisions observed in either neural crest cells [20] or healthy and metastatic epithelial cells [19, 22]. Tuning only the four parameters of cell-cell adhesion *σ*, critical overlap *O*_*crit*_ and the strengths of contact/front repolarization *k*_*CR*_ and *k*_*FR*_ is enough to have a very high rate of each outcome. We also have identified a set of parameters that yields all of the cases seen in neural crest cells, with rates in good agreement with those seen by [20]. We are also able to reproduce additional qualitative aspects of experimental collisions. This includes the peak in the velocity for the reversal case (S1 Fig), and the presence of “cell trains” (Fig 9), which Desai et al. reported in [19] for NRK-52E cells.

**Fig 10:**
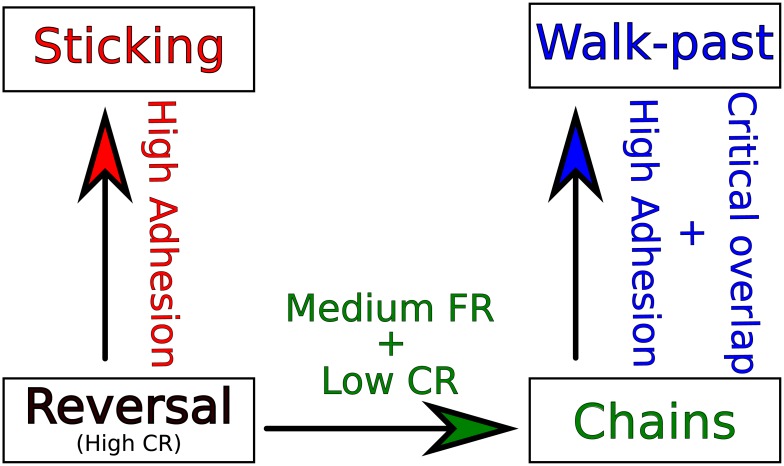
Schematic picture of parameters controlling the different outcomes.

The transition between these states in our model is summarized in Fig 10, which emphasizes that multiple parameters must be changed to control the different outcomes. If contact repolarization is strong (e.g. *k*_*CR*_ ≥ 0.03*s*^−1^, Fig 7) together with a weaker adhesion strength (*σ* ≤ 3.84*σ*_0_, Fig 4), we can create a 100% reversal rate. Increasing the adhesion above this value yields a sharp transition to a high sticking rate. If instead of increasing adhesion from the 100% reversal rate parameters, we reduce the contact repolarization and tune the front repolarization to a medium value (*k*_*FR*_ ∼ 0.05*s*^−1^, Fig 7), we can robustly create chain events. Increasing the adhesion and introducing a nonzero critical overlap then yields a high walk-past rate—though this requires some degree of tuning (Figs 5 and 6).

We also make predictions for how the rates of walk-past depend on the stripe width. We find that the walk-past rate is at its maximum of 99% for *d* = 26*μm* (Fig 8). For narrower channels it drops rapidly to zero, as the cells can no longer squeeze past each other. Surprisingly, wider stripes (*d* = 30*μm*) can also reduce the walk-past rate for some parameter combinations. This occurs when the absence of confinement allows the cells an additional route to separate prior to undergoing walk-past, leading to more reversals and fewer walk-past cases. Interestingly, [22] have also found that walk-past rates can be non-monotonic in stripe width for metastatic MDA-MB-231 mammary epithelial cells, though not for nontransformed MCF-10A epithelial cells. However, they see an opposite behavior to our simulations—with stripes of minimum width leading to a minimum in cell walk-past.

We report a set of parameters that creates a mix of outcomes similar to those seen experimentally by [20], *k*_*FR*_ = 0.04*s*^−1^, *k*_*CR*_ = 0.01*s*^−1^, *O*_*crit*_ = 0.15*μm*^−2^ and *σ* = 3.85*σ*_0_. With these parameters we observe 69% reversals, 27% sticking, 4% walk-past, and no chaining outcomes. These results, though, can be highly sensitive to small variations in parameters (Table 2, S3 Fig), similar to the sensitivity to adhesion that occurs near the sticking-reversal transition (Fig 4). However, the most sensitive parameters are not necessarily the expected ones. Variations in parameters that control the biochemical interactions (*k*_*CR*_ and *k*_*FR*_) must be large (≥ 50%) to have an effect. Contrarily, the cells are very sensitive to changes in mechanical parameters that affect the sticking transition (*α*, *σ*, contraction strength *β* and line tension *γ*). Here, even small variations of 1% or less can switch collisions from majority reversal to majority sticking. This high sensitivity of the cells tells us that either a) cells are very carefully tuned towards the sticking transition, or b) cell-to-cell variations or variability of effects on contact play a major role in the coexistence of reversal and sticking—those cells that reverse may have lower cadherin expression levels or other biochemical differences. We argue that b) is more likely: typical scales of variation in, e.g. protein expression levels are 10% and higher [50, 51], and eukaryotic cell responses to signals may also vary significantly from cell to cell [52, 53]. Cell-to-cell variations would be expected to significantly increase the robustness of certain types of responses. For instance, even if the sticking-reversal transition is sharp, as in Fig 4, if there is a population of high-adhesion cells and a population of low-adhesion cells, sticking will occur when two high-adhesion cells stick; this will not depend on the precise value of *σ* for the high-adhesion cells.

Our simulations use, as a central hypothesis, the idea that cell-cell contact leads to the generation of a chemical that inhibits a polarity protein, taken to be Rac, which we view as a reasonable first hypothesis to model CIL in neural crest [25]. What do we gain from modeling cell interactions to this level of biochemical detail, rather than using more generic mechanisms like flocking [5, 54], “velocity alignment” [55–57] or others [9, 37, 58–61]? Some of our results are qualitatively surprising in comparison to those that would be expected from minimal models. With a minimal model (e.g. our simplified model of CIL in [9]), we would intuitively expect cell-cell adhesion to monotonically reduce walk-past. However, we find that increasing adhesion can promote walk-past events (Fig 5). This arises from the interaction of adhesion, which influences the cell-cell contact area, with the inhibitor generated at contact. We also note the importance of the different thresholds at which contact and front repolarization arise (the critical overlap). Neither of these effects would arise in a simplified model, and we argue that, for a full understanding of cell-cell interactions, modeling biochemical interactions like CIL and cell contact dynamics are both necessary.

Our results show that the coupling of detailed cell shape dynamics with biochemistry can lead to unintuitive behaviors, like the nonmonotonic effects of adhesion. This illustrates the importance of complementing minimal models of cells as self-propelled particles [5, 9] with increasingly detailed models for cell shape and mechanics. The influence of cell shape and adhesion on collective cell migration has also recently been studied in the context of the jamming transition in epithelial monolayers [62]; our results suggest that combining signaling with shape may lead to new effects.

In partial contrast with our results, recent experiments studying the interaction of pairs of mammary epithelial cells colliding on micropatterns have found that increasing E-cadherin expression decreases the odds of walk-past [22]. These data suggest that either modeling the interaction of epithelial cells requires more than our straightforward CIL mechanism, or that only one portion of a non-monotonic dependence on E-cadherin expression has been explored for these systems.

Many extensions of our modeling may be interesting. One natural choice would be to develop a more detailed description of the statistics of cell protrusions in neural crest, e.g. including stochastic protrusion and retraction [63] as has been modeled for Dictyostelium [64, 65]. Inclusion of hydrodynamic flow within the cells, as we have previously studied for single cells [31, 41], could potentially be relevant as well, especially if we were to extend our study from collisions in micropatterns to microchannels [66]. Another feature that we have not yet included in our modeling is the secretion of chemoattractant and subsequent chemotaxis to this signal, or “co-attraction,” which is known to play a role in promoting the cohesion of neural crest cells [67]; this could be included in our model by techniques along the lines of recent approaches [10, 60, 61, 68]. However, the graded response to a chemoattractant across a cell is itself an area of a great deal of interesting research [64, 65, 69–72], and including this feature could lead to significant additional complexity.

What can we understand about experiments studying collisions of neural crest cells [20] in the context of our simulations? Scarpa et al. did not report any chaining events. We can eliminate chains by either making the front repolarization rate high, or including a contact repolarization effect. Even a weak contact repolarization can dominate a collision and prevent chains, especially when front repolarization is also present (Fig 7). The walk-past case shows that a nonzero CR is necessary for the cells to separate after passing each other. However, the CR cannot be too strong without suppressing walk-past (Fig 6). Taken together, this suggest that in modeling neural crest cell collisions, we should assume both FR and CR are present, with FR stronger than the CR.

From these results, we reach three broad conclusions. First, based on our model, we would propose that treatments that increase sticking are likely to have effects either on cell-cell adhesion (e.g. cadherin expression levels) or on single-cell properties (e.g. changing cell speed or contractility). Secondly, because in order to create the experimental mix of outcomes from a simulation, we needed to carefully tune parameters, we suggest that the range of outcomes in [20] may arise more from cell-to-cell variation than it does from the stochastic motion of individual cells. Third, we predict that altering either cell-cell adhesion or micropattern size can lead to non-monotonic changes in outcome frequency.

## Supporting Information

S1 Movie Strong contact repolarization results in a reversal. Parameters: *k*_*CR*_ = 0.025*s*^−1^, *k*_*FR*_ = 0.075*s*^−1^, *O*_*crit*_ = 0*μm*^−2^, *σ* = 2.25*σ*_0_.
(MP4)

S2 Movie Strong adhesion leads to a pair of sticking cells. Parameters: *k*_*CR*_ = 0.05*s*^−1^, *k*_*FR*_ = 0.0*s*^−1^, *O*_*crit*_ = 0*μm*^−2^, *σ* = 4.2*σ*_0_.
(MP4)

S3 Movie Adhesion and a tuned balance of FR and CR enables walk-past. Parameters: *k*_*CR*_ = 0.004*s*^−1^, *k*_*FR*_ = 0.075*s*^−1^, *O*_*crit*_ = 0.15*μm*^−2^, *σ* = 3.7*σ*_0_.
(MP4)

S4 Movie Tuning FR creates chains. Parameters: *k*_*CR*_ = 0.0025*s*^−1^, *k*_*FR*_ = 0.05*s*^−1^, *O*_*crit*_ = 0.0*μm*^−2^, *σ* = 2.25*σ*_0_.
(MP4)

S5 Movie Strong adhesion with FR creates persistently rotating pairs. Parameters: *k*_*CR*_ = 0*s*^−1^, *k*_*FR*_ = 0.05*s*^−1^, *O*_*crit*_ = 0.15*μm*^−2^, *σ* = 4*σ*_0_.
(MP4)

S6 Movie Without biochemical interactions cell-cell collisions do not always resemble any of the four experimental cases. Outcomes can be ambiguous with repeated loss of polarization and spontaneous repolarization. Parameters: *k*_*CR*_ = 0*s*^−1^, *k*_*FR*_ = 0*s*^−1^, *O*_*crit*_ = 0*μm*^−2^, *σ* = 2.25*σ*_0_.
(MP4)

S7 Movie For weak FR cells migrate as a chain before the walk past each other. Parameters: *k*_*CR*_ = 0*s*^−1^, *k*_*FR*_ = 0.05*s*^−1^, *O*_*crit*_ = 0.15*μm*^−2^, *σ* = 4*σ*_0_.
(MP4)

S8 Movie A strong CR yields cells that are well distributed on the stripe. Parameters: *k*_*CR*_ = 0.25*s*^−1^, *k*_*FR*_ = 0*s*^−1^, *O*_*crit*_ = 0*μm*^−2^, *σ* = 2.25*σ*_0_.
(MP4)

S9 Movie Strong adhesion results in several pairs of sticking cells. Parameters: *k*_*CR*_ = 0.25*s*^−1^, *k*_*FR*_ = 0*s*^−1^, *O*_*crit*_ = 0*μm*^−2^, *σ* = 4*σ*_0_.
(MP4)

S10 Movie For walk-past parameters, cells can form trains which break up shortly after. Parameters: *k*_*CR*_ = 0.004*s*^−1^, *k*_*FR*_ = 0.075*s*^−1^, *O*_*crit*_ = 0.15*μm*^−2^, *σ* = 3.75*σ*_0_.
(MP4)

S11 Movie For chain parameters cells form persistent trains. Parameters: *k*_*CR*_ = 0*s*^−1^, *k*_*FR*_ = 0.05*s*^−1^, *O*_*crit*_ = 0*μm*^−2^, *σ* = 2.25*σ*_0_.
(MP4)

S1 Fig Speed trace of cells during typical reversal event. Comparison of the velocity between the experiments (right, figure from [20], licensed under CC-BY) and our simulations (left) for reversal events. Our simulation shows a typical course of the center of mass velocity. Used parameters in the simulation: *α* = 0.4*α*_0_, *k*_*CR*_ = 0.02*s*^−1^, *k*_*CR*_ = 0.075*s*^−1^, *O*_*crit*_ = 0*μm*^−2^ and *σ* = 2.25*σ*_0_.
(PDF)

S2 Fig Dependence of sticking transition on length of simulation. Left: 50% contour line of the sticking/reversal transition for two different observation times (compare with Fig 4), right: dependence of the sticking percentage on *k*_*CR*_ for different simulation length. Parameters used in the simulation: *k*_*FR*_ = 0*s*^−1^, *O*_*crit*_ = 0*μm*^−2^ (both), *k*_*CR*_ = 0.1*s*^−1^ (left), *α* = 0.4*α*_0_ and *σ* = 3.86*σ*_0_ (right).
(PDF)

S3 Fig Full parameter variation. With parameters *k*_*FR*_ = 0.04*s*^−1^, *k*_*CR*_ = 0.01*s*^−1^, *O*_*crit*_ = 0.15*μm*^−2^, *σ* = 3.85*σ*_0_, *α* = 0.4*α*_0_, *β* = 0.2*pN*/*μm*, *γ* = 1.8*pN* and *k*_*b*_ = 10*s*^−1^. Entries in red are the parameters which reproduce the experimental outcome.
(EPS)

S1 Table Parameter Table. (PDF)

S2 Table Automatic detection of the collision outcome. (PDF)

S1 Text Supplemental Information. S1 Text provides the following: i) a comparison of the cell speed in a reversal event between experiment and simulation ii) Dependence of the sticking transition on length of simulation iii) Full output of the parameter variation around the parameter set which reproduces the experimental outcome iv) numerical details v) A table with the default parameters.
(PDF)

S1 Data Supplemental Information. S1 Data provides the data for all figures in the paper and in S1 Text.
(ZIP)
