# Supplementary figures and images for: Modeling Contact Inhibition of Locomotion of Colliding Cells Migrating on Micropatterned Substrates
Source: PLoS Comput Biol. 2016 Dec 16;12(12):e1005239. doi: 10.1371/journal.pcbi.1005239 (PMC5161303; doi:10.1371/journal.pcbi.1005239)

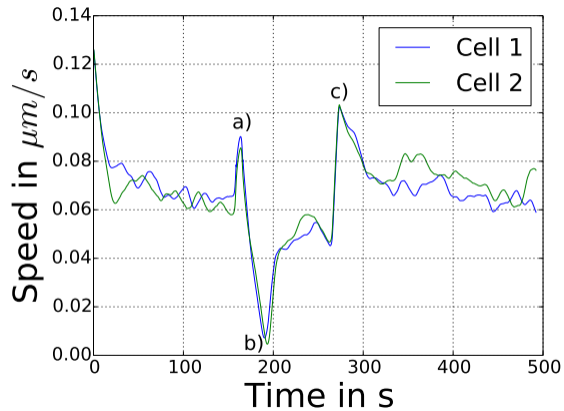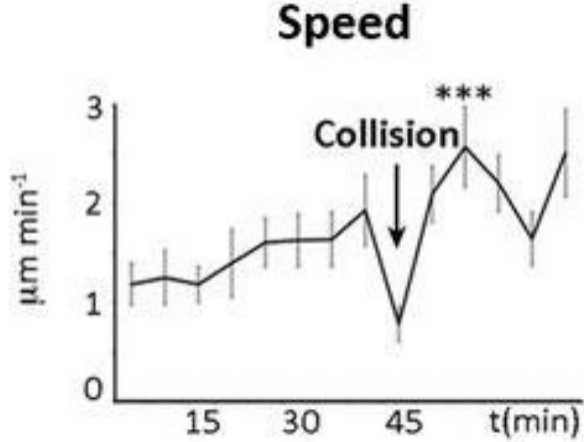

Supplement: S1 Fig — Comparison of the velocity between the experiments (right, figure from [20], licensed under CC-BY) and our simulations (left) for reversal events. Our simulation shows a typical course of the center of mass velocity. Used parameters in the simulation: α = 0.4α0, kCR = 0.02s−1, kCR = 0.075s−1, Ocrit = 0μm−2 and σ = 2.25σ0. (PDF) [file pcbi.1005239.s012.pdf]

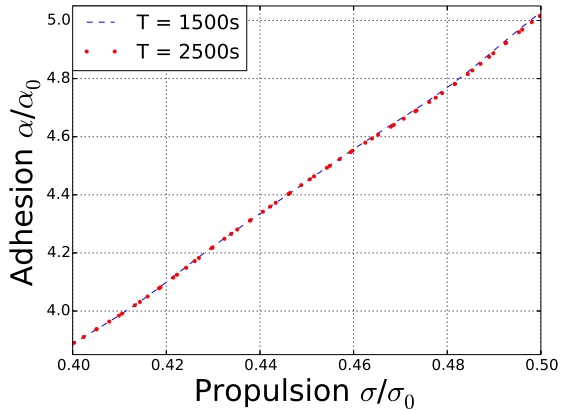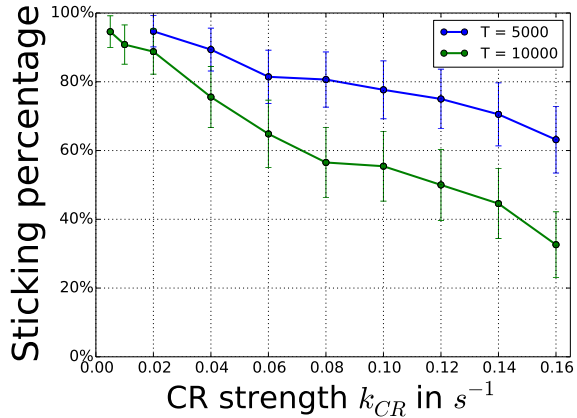

Supplement: S2 Fig — Left: 50% contour line of the sticking/reversal transition for two different observation times (compare with Fig 4), right: dependence of the sticking percentage on kCR for different simulation length. Parameters used in the simulation: kFR = 0s−1, Ocrit = 0μm−2 (both), kCR = 0.1s−1 (left), α = 0.4α0 and σ = 3.86σ0 (right). (PDF) [file pcbi.1005239.s013.pdf]
