# Supplementary material for: Modeling Contact Inhibition of Locomotion of Colliding Cells Migrating on Micropatterned Substrates
Source: PLoS Comput Biol. 2016 Dec 16;12(12):e1005239. doi: 10.1371/journal.pcbi.1005239 (PMC5161303; doi:10.1371/journal.pcbi.1005239)
Supplement: S1 Table — (PDF) [file pcbi.1005239.s015.pdf]

Table 1: **Table of default simulation parameters**

Parameter	Description	Value
α	Protrusion coefficient	0.4 pN / μm^3
β	Retraction coefficient	0.2 pN / μm
γ	Cell tension coefficient	1.8 pN
κ	Cell bending coefficient	5 pN μm^2
g	Cell-cell body repulsion coefficient	1 pN/ μm
R_{cell}	Initial radius of cell	9 μm
τ	Friction coefficient	2.62 pN s / μm^2
ϵ	Phase field width	2 μm
k_a	Unitless base activation rate	0.01
k_b	Overall activation rate	10 s^{-1}
k_c	Deactivation rate	10 s^{-1}
K_a	Positive feedback threshold for actin promoter (Rho GTPase) concentration	1 μm^{-2}
D_ρ	Actin promoter (Rho GTPase) diffusion coefficient	0.8 μm^2/s
N_{tot}	Total amount of actin promoter (unitless)	800
D_I	Inhibitor diffusion coefficient	0.5 μm^2/s
k_{-I}	Degradation rate of I	0.2 s^{-1}
ρ^{char}	characteristic scale of ρ	1 μm^{-2}
η	Noise intensity	0.02 $\mu m/s$
d	Stripe width	26 μm
Δt	Numerical time step	0.004 s

These parameters are used throughout the paper; any deviation from them is explicitly noted.
