# Supplementary material for: Modeling Contact Inhibition of Locomotion of Colliding Cells Migrating on Micropatterned Substrates
Source: PLoS Comput Biol. 2016 Dec 16;12(12):e1005239. doi: 10.1371/journal.pcbi.1005239 (PMC5161303; doi:10.1371/journal.pcbi.1005239)
Supplement: S2 Table — (PDF) [file pcbi.1005239.s016.pdf]

Table 1: **Definitions of the different cases**

not included	a cell turns around due to internal noise (distance between the cells is greater than $3 \times r_{cell}$ when a cell turns)
rotation	cells turn around **and** pass each other more than two times
sticking	y-velocity of both cells is below $0.005 \mu m/s$ **or** the distance between the cells is smaller than $20m$ for the second half of the simulation and both cells turn around **or** the distance between the cells is smaller than $35m$ and the y-velocity of both cells is below $0.02 \mu m/s$
walk-past	no cell turns around **or** both cells turn around and pass each other two times over the course of the simulation ("double walk-past") **and** the distance between the cells is greater than $3 \times r_{cell}$ when the simulation ends.
reversal	both cells turn around **and** the distance between the cells is greater than $3 \times r_{cell}$ when the simulation ends **and** the cells have never passed each other.
chain	only one cell turns around **and** the distance between the cells is less than $3 \times r_{cell}$ when the simulation ends **and** the cells have never passed each other.

A turn around is detected if the y-velocity of a cell changes its sign. For the latter three cases we only compare the sign of the start and the end velocity to detect a turn around. To compute the rates for each case we divide the count for a specific outcome by the total number of simulation, excluding the first entry of the table ("not included").
