# Supplementary material for: Modeling Contact Inhibition of Locomotion of Colliding Cells Migrating on Micropatterned Substrates
Source: PLoS Comput Biol. 2016 Dec 16;12(12):e1005239. doi: 10.1371/journal.pcbi.1005239 (PMC5161303; doi:10.1371/journal.pcbi.1005239)
Supplement: S1 Text — provides the following: i) a comparison of the cell speed in a reversal event between experiment and simulation ii) Dependence of the sticking transition on length of simulation iii) Full output of the parameter variation around the parameter set which reproduces the experimental outcome iv) numerical details v) A table with the default parameters. (PDF) [file pcbi.1005239.s017.pdf]

Supplementary Information: Modeling Contact Inhibition of Locomotion of Colliding Cells Migrating on Micropatterned Substrates

Dirk Alexander Kulawiak Brian A. Camley Wouter-Jan Rappel

1 Speed trace of cells during typical reversal event

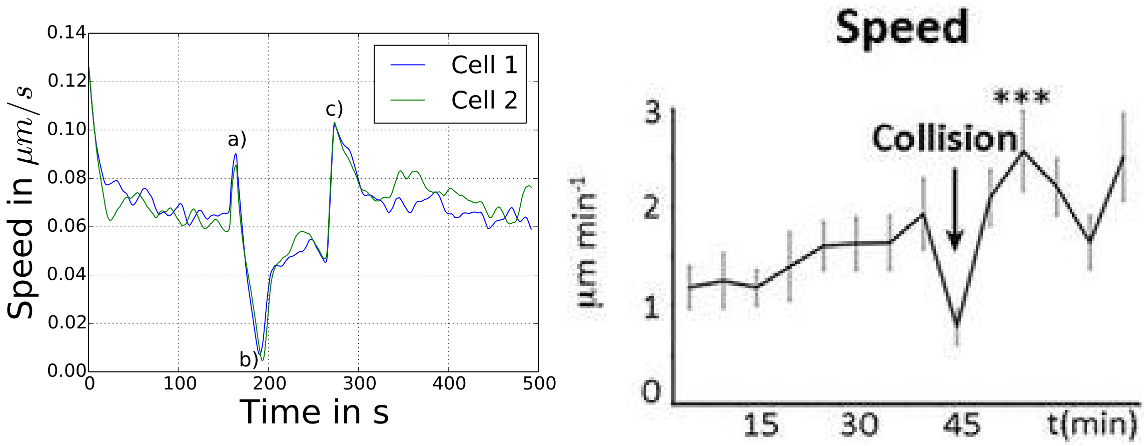

Figure S1: Comparison of the velocity between the experiments (right, figure from Ref. [20] in the main text, licensed under CC-BY) and our simulations (left) for reversal events. Our simulation shows a typical course of the center of mass velocity. Used parameters in the simulation: $\alpha = 0.4\alpha_0$, $k_{CR} = 0.02s^{-1}$, $k_{CR} = 0.075s^{-1}$, $O_{crit} = 0\mu m^{-2}$ and $\sigma = 2.25\sigma_0$.

2 Dependence of sticking transition on length of simulation

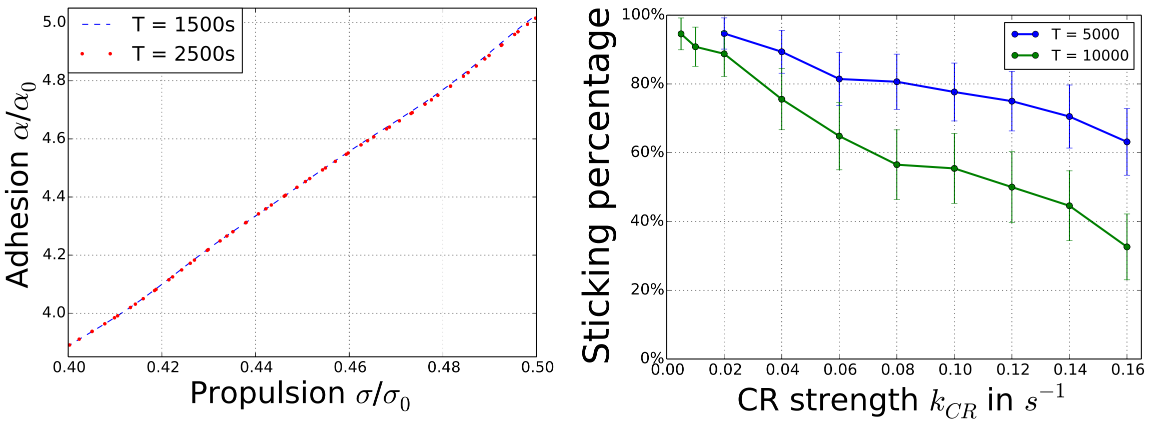

Figure S2: Left: 50% contour line of the sticking/reversal transition for two different observation times (compare with Figure 4), right: dependence of the sticking percentage on k_{CR} for different simulation length. Parameters used in the simulation: $k_{FR} = 0s^{-1}$, $O_{crit} = 0\mu m^{-2}$ (both), $k_{CR} = 0.1s^{-1}$ (left), $\alpha = 0.4\alpha_0$ and $\sigma = 3.86\sigma_0$ (right).

In Figure S2 we show the dependence of the sticking percentage on the simulation length for two different cases. We want to demonstrate that changes of the simulation length can a) change the collision behavior quantitatively near the reversal-sticking transition, but b) do in general not qualitatively change the outcome.

In the left panel we show the 50% contour line of the sticking/reversal transition for two different simulation lengths. Here, half of the of the collisions result in a pair of sticking cells and the other half ends in a reversal. With shorter simulation times the 50% contour line is shifted to a very slightly lower adhesion strength. The critical qualitative characteristics also stay the same: Increasing the propulsion strength α moves the sticking/reversal transition to larger adhesion strengths.

In the right panel of Figure S2 we show how changes in k_{CR} affect the sticking percentage, and how this is affected by simulation length. This effect only occurs near the sticking-reversal transition. In agreement with our analysis of parameter variations (see Table 1 and Figure S3), increasing k_{CR} can decrease the percentage of pairs that stick. Though the effect of longer simulation times is stronger here, the qualitative behavior remains the same for both simulation times. In longer simulations more cells detach and the sticking percentage decreases and for both simulation lengths, the sticking percentage increases for lower values of k_{CR}.

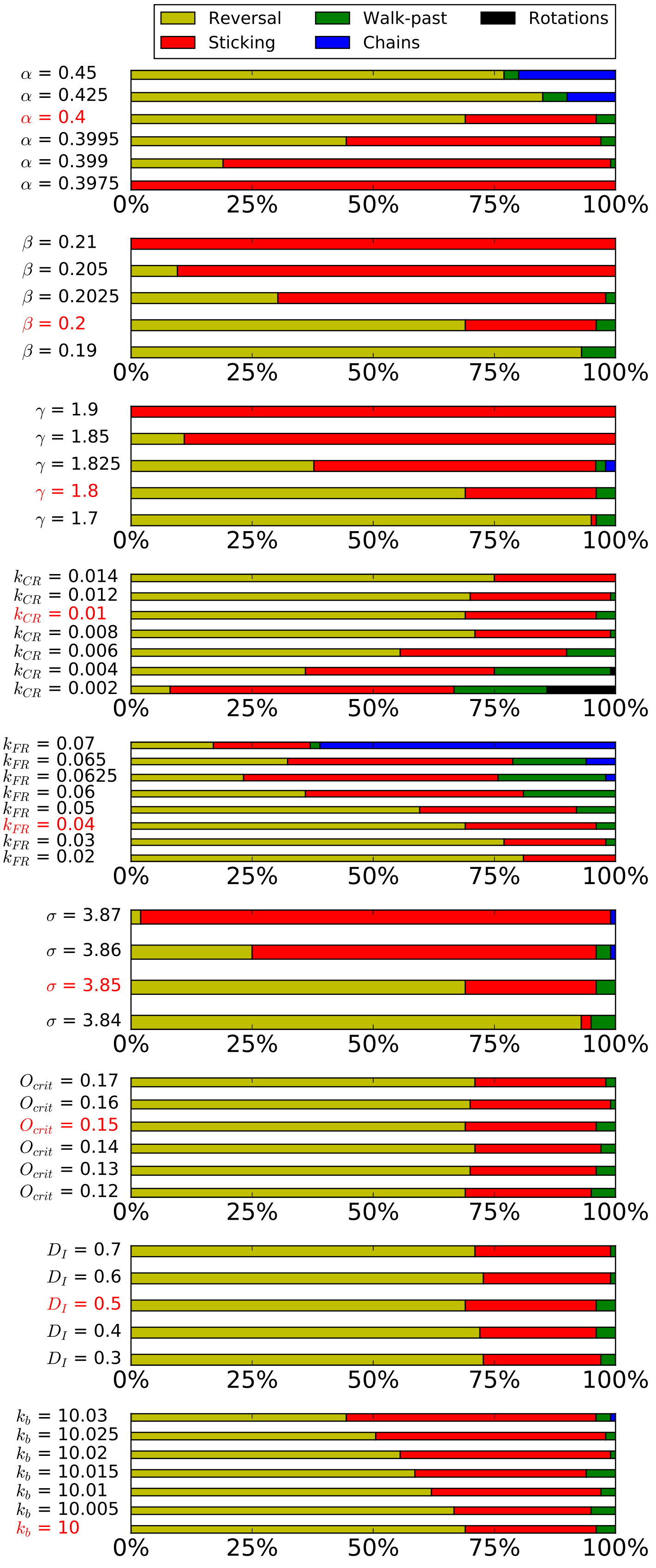

Figure S3: Full parameter variation with $k_{FR} = 0.04s^{-1}$, $k_{CR} = 0.01s^{-1}$, $O_{crit} = 0.15\mu m^{-2}$, $\sigma = 3.85\sigma_0$, $\alpha = 0.4\alpha_0$, $\beta = 0.2pN/\mu m$, $\gamma = 1.8pN$ and $k_b = 10s^{-1}$. Entries in red are the parameters which reproduce the experimental outcome.

Table S1: **Table of default simulation parameters**

Parameter	Description	Value
α	Protrusion coefficient	0.4 pN / μm^3
β	Retraction coefficient	0.2 pN / μm
γ	Cell tension coefficient	1.8 pN
κ	Cell bending coefficient	5 pN μm^2
g	Cell-cell body repulsion coefficient	1 pN/ μm
R_{cell}	Initial radius of cell	9 μm
τ	Friction coefficient	2.62 pN s / μm^2
ϵ	Phase field width	2 μm
k_a	Unitless base activation rate	0.01
k_b	Overall activation rate	10 s^{-1}
k_c	Deactivation rate	10 s^{-1}
K_a	Positive feedback threshold for actin promoter (Rho GTPase) concentration	1 μm^{-2}
D_ρ	Actin promoter (Rho GTPase) diffusion coefficient	0.8 μm^2/s
N_{tot}	Total amount of actin promoter (unitless)	800
D_I	Inhibitor diffusion coefficient	0.5 μm^2/s
k_{-I}	Degradation rate of I	0.2 s^{-1}
ρ^{char}	characteristic scale of ρ	1 μm^{-2}
η	Noise intensity	0.02 $\mu m/s$
d	Stripe width	26 μm
Δt	Numerical time step	0.004 s

These parameters are used throughout the paper; any deviation from them is explicitly noted.

3 Supplemental Methods

We solve the equations of motion on a large rectangular domain of size $L \times L$ ($L = 100\mu m$) with periodic boundary conditions. To save computation time, each cell has its own computation box with the size $L_{small} \times L_{small}$ ($L_{small} = 50\mu m$) and periodic boundary conditions, in which we solve the phase field and reaction-diffusion equations. This box is recentered if the cell migrates close to the boundary. Then, the small box is shifted in the larger domain. The values of the newly created points are assigned by a periodic shift. Since the cell does not come close to the boundaries we assume that all fields (ρ,I,ϕ) are zero outside of the box.

The computational boxes for the individual cells are always centered around the center of mass (COM) of a cell, where the COM is approximated to the nearest grid point of the larger grid. Both types of grid have the same properties, i.e. they are both regular, rectangular grids, with the same $\Delta x = \Delta y$. This has the consequence that each point of a computational boxes can be mapped clearly and without any interpolation/approximation to a grid point in the larger grid. When the equations of motion involve terms of two different cells (for example $\sum_{i \neq j} \phi^{(i)}(\mathbf{r}, t)\phi^{(j)}(\mathbf{r}, t)$), we transfer the required values from each smaller box to the larger grid from where they can be mapped back to every small box.

We solve the phase field equation with a semi-implicit spectral method to compute the first derivative for the advection and adhesion terms, the second derivative for the line tension and the fourth derivative for the bending term. All other terms and the reaction-diffusion equations are handled explicitly. Below, we introduce the substitutes $I \equiv I(\mathbf{r}, t)$ and $\phi \equiv \phi(\mathbf{r}, t)$. If those quantities are used at different locations or times, we will explicitly state the dependence.

The phase field equations for each cell

$$\begin{aligned}
\partial_t \phi =& \frac{1}{\tau} \left(\alpha \rho(\mathbf{r}, t) \chi(\mathbf{r}) - \beta \right) |\nabla \phi| + \frac{\gamma}{\tau} \left[\nabla^2 \phi - \frac{G'(\phi)}{\epsilon^2} \right] - \frac{\kappa}{\tau} \left[\nabla^2 - \frac{G''(\phi)}{\epsilon^2} \right] \left[\nabla^2 \phi - \frac{G'(\phi)}{\epsilon^2} \right] \\
=& \frac{1}{\tau} \left(\alpha \rho(\mathbf{r}, t) \chi(\mathbf{r}) - \beta \right) |\nabla \phi| + \frac{\gamma}{\tau} \nabla^2 \phi - \frac{\kappa}{\tau} \nabla^4 \phi \\
& - \frac{\gamma}{\tau} \frac{G'(\phi)}{\epsilon^2} + \frac{\kappa}{\tau} \left[\nabla^2 \frac{G'(\phi)}{\epsilon^2} + \frac{G''(\phi)}{\epsilon^2} \nabla^2 \phi - \frac{G'(\phi) G''(\phi)}{\epsilon^4} \right]
\end{aligned} \tag{S1}$$

are solved semi-implicitly

$$\phi(\mathbf{r}, t + \Delta t) - \frac{\Delta t \gamma}{\tau} \nabla^2 \phi(\mathbf{r}, t + \Delta t) + \frac{\Delta t \kappa}{\tau} \nabla^4 \phi(\mathbf{r}, t + \Delta t) = \phi(\mathbf{r}, t) + \Delta t F_{exp}. \tag{S2}$$

Time-stepping in Fourier space leads to

$$\phi(\mathbf{q}, t + \Delta t) = \frac{\phi(\mathbf{q}, t) + \Delta t \{F_{exp}(t)\}_q}{1 + (\Delta t \gamma / \tau)|\mathbf{q}|^2 + (\Delta t \kappa / \tau)|\mathbf{q}|^4}, \tag{S3}$$

where $\{...\}_q$ indicates the Fourier transform and the explicit part

$$F_{exp} = \frac{1}{\tau} \left(\alpha \rho(\mathbf{r}, t) \chi(\mathbf{r}) - \beta \right) |\nabla \phi| - \frac{\gamma}{\tau} \frac{G'(\phi)}{\epsilon^2} + \frac{\kappa}{\tau} \left[\nabla^2 \frac{G'(\phi)}{\epsilon^2} + \frac{G''(\phi)}{\epsilon^2} \nabla^2 \phi - \frac{G'(\phi) G''(\phi)}{\epsilon^4} \right] \tag{S4}$$

is evaluated on the rectangular grid at time t. The derivatives in Eq. (S4) are computed using spectral methods.
In the multi-cell case, the phase field equation is supplemented by a term from the functional derivative of the cell-cell interaction energy, which is handled explicitly. In calculating this term, we use a 17-point finite difference stencil. This is to avoid lattice artifacts, like cell-cell interfaces favoring fixed directions with respect to the axis. Details of the derivation can be found in Section 4.

The stochastic reaction-diffusion equation for the inhibitor I

$$\partial_t (\phi I) = \nabla [\phi D_I \nabla I] + \phi f_I[I, \{\phi\}, ..] + \xi(\mathbf{r}, t) \tag{S5}$$

is solved explicitly with a Euler-Maruyama method

$$\frac{\phi(\mathbf{r}, t + \Delta t) - \phi(\mathbf{r}, t)}{\Delta t} I + \frac{I(\mathbf{r}, t + \Delta t) - I(\mathbf{r}, t)}{\Delta t} \phi = \nabla [\phi D_I \nabla I] + \phi f_I[I, \{\phi\}, ..] + \Xi(\mathbf{r}, t) \tag{S6}$$

leading to

$$I(\mathbf{r}, t + \Delta t) = \frac{2\phi(\mathbf{r}, t) - \phi(\mathbf{r}, t + \Delta t)}{\phi} I + \Delta t \frac{\nabla [\phi D_I \nabla I]}{\phi} + \Delta t f_I[I, \{\phi\}, ..] + \Xi(\mathbf{r}, \Delta t). \tag{S7}$$

The Gaussian random variable $\Xi(\mathbf{r}, t) = \int_t^{t+\Delta t} dt' \xi(\mathbf{r}, t')$ has zero mean and variance of

$$\langle \Xi(\mathbf{r}, \Delta t), \Xi(\mathbf{r}', \Delta t) \rangle = \eta^2 \Delta t \delta(\mathbf{r} - \mathbf{r}'). \tag{S8}$$

Furthermore, we assume the noise to be uncorrelated between lattice sites and in time. To make sure that the strength of the noise is independent of the grid and time resolution we normalize it by $\sqrt{\Delta t}/\Delta x$. In our simulations we compute $\Xi(\mathbf{r}, \Delta t) = Y\sqrt{\eta^2 \Delta t/\Delta x^{-2}}$, with Y being a Gaussian distributed random number with zero mean and variance one.

Dividing by ϕ in Eq. (S7) is obviously a numerical issue when $\phi \ll 1$. Following the usual method for phase field equations Ref. [46] in the main text, we only apply this equation above a threshold value, $\phi \geq 10^{-4}$. For $\phi < 10^{-4}$, we solve this equation, but multiplied by ϕ; this serves as a numerical convenience to reduce I and ρ where $\phi \ll 1$ and thus outside of the region where the reaction-diffusion equations would be solved in the sharp interface limit.

In particular, the diffusion term in Eq. (S7) $\nabla[\phi D_I \nabla I]$ is computed explicitly with a half-point stepping. In one dimension at time t and grid point i it has the form

$$\nabla[\phi D_I \nabla I]_i = D_I \left(\frac{\phi_{i+1} + \phi_i}{2} \frac{I_{i+1} - I_i}{\Delta x} - \frac{\phi_{i-1} + \phi_i}{2} \frac{I_i - I_{i-1}}{\Delta x} \right) /\Delta x. \quad \text{(S9)}$$

The equation for $\rho(\mathbf{r}, t)$ is solved identical, it only lacks the noise $\xi(\mathbf{r}, \Delta t)$.
We use OpenCL to solve the equations on GPUs. To compute the FFT needed for the spectral methods, we use clFFT in batch mode.

Initial conditions In our simulations, we confine the cells to stripes with width $d = 26\mu m$ unless otherwise noted. The cells are initially separated by $40\mu m$, but polarized toward one another. For each parameter combination we conducted 100 simulations, if not stated otherwise. To reduce the variability between runs with different parameters, we use the same seed to create the random numbers for the same run. Of course we never reuse a seed for runs within the same parameters. All default parameters are shown in S1 Table.

Automatic detection of the collision outcome To automatically detect the outcome in our simulations we are defining the different events by the rules given in Table S2. It should be noted that the different possible results are checked in the order they are listed in Table S2. As soon as the conditions of one case apply the others are not checked anymore. Additionally, the simulations were aborted when the cells had a distance of $50\mu m$ between them (for reversal and walk-past cases) or they migrated $100\mu m$ in the same direction (chain). For the sticking case the simulation times are discussed in the relevant section.

Table S2: **Definitions of the different cases**

not included	a cell turns around due to internal noise (distance between the cells is greater than $3 \times r_{cell}$ when a cell turns)
rotation	cells turn around **and** pass each other more than two times
sticking	y-velocity of both cells is below $0.005\mu m/s$ **or** the distance between the cells is smaller than $20m$ for the second half of the simulation and both cells turn around **or** the distance between the cells is smaller than $35m$ and the y-velocity of both cells is below $0.02\mu m/s$
walk-past	no cell turns around **or** both cells turn around and pass each other two times over the course of the simulation ("double walk-past") **and** the distance between the cells is greater than $3 \times r_{cell}$ when the simulation ends.
reversal	both cells turn around **and** the distance between the cells is greater than $3 \times r_{cell}$ when the simulation ends **and** the cells have never passed each other.
chain	only one cell turns around **and** the distance between the cells is less than $3 \times r_{cell}$ when the simulation ends **and** the cells have never passed each other.

A turn around is detected if the y-velocity of a cell changes its sign. For the latter three cases we only compare the sign of the start and the end velocity to detect a turn around. To compute the rates for each case we divide the count for a specific outcome by the total number of simulation, excluding the first entry of the table ("not included").

4 Derivation of higher order stencil for adhesion term

In this section we derive a higher order finite-differences stencil to compute the adhesion term for multiple cells

$$\frac{\delta H_{adh}}{\delta \phi} = -\frac{\delta}{\delta \phi} \sum_{i \neq j} \int d^2 r \frac{\sigma \epsilon^3}{4} |\nabla \phi^{(i)}|^2 * |\nabla \phi^{(j)}|^2 = -\sigma \epsilon^3 \nabla (f * \nabla \phi). \quad \text{(S10)}$$

Here, $f = \sum_{i \neq j} |\nabla \phi^{(j)}|^2$, which is calculated using spectral methods.
Eq. (S10) is solved with the finite-difference

$$\partial_x \to h^{-1} \delta, \quad \text{(S11)}$$

where h is the lattice spacing and

$$\delta u_i \equiv u_{i+1/2} - u_{i-1/2}, \tag{S12}$$

is the centered finite-difference. For stability reasons Eq. (S10) is solved with a high order finite-differences scheme. One way to do this is to use the series expansion (F.B. Hildebrand's Numerical Analysis, Eq. 5.3.12):

$$\partial_x = \frac{2}{h}\sinh^{-1}\frac{\delta}{2} = \frac{1}{h}\left[\delta - \frac{1}{24}\delta^3 + \cdots\right]. \tag{S13}$$

If we apply this to $\partial_x(f\partial_x\phi)$, we find

$$\partial_x(f\partial_x\phi) \to \frac{1}{h^2}\left[\delta(f\delta\phi) - \frac{1}{24}\delta^3 f\delta\phi - \frac{1}{24}\delta f\delta^3\phi + \cdots\right] \tag{S14}$$

when we omit all terms $O(\delta^5)$ or higher. The terms in Eq. (S14) in one dimension at time t and grid point i evaluate to:

$$\begin{aligned}\delta\left[f_i(\delta\phi_i)\right] &= \left(\delta\left[f_i(\phi_{i+1/2} - \phi_{i-1/2})\right]\right) \\ &= \left[f_{i+1/2}(\phi_{i+1} - \phi_i) - f_{i-1/2}(\phi_i - \phi_{i-1})\right].\end{aligned} \tag{S15}$$

The points which are of the grid are computed with linear interpolation, yielding

$$\delta\left[f_i(\delta\phi_i)\right] = \frac{f_{i+1} + f_i}{2}(\phi_{i+1} - \phi_i) - \frac{f_{i-1} + f_i}{2}(\phi_i - \phi_{i-1}), \tag{S16}$$

$$\begin{aligned}\delta^3 f_i\delta\phi_i &= \delta^2(f_{i+1/2} - f_{i-1/2})\delta\phi \\ &= \delta(f_{i+1} - 2f_i + f_{i-1})\delta\phi \\ &= (f_{i+3/2} - 3f_{i+1/2} + 3f_{i-1/2} - f_{i-3/2})(\phi_{i+1/2} - \phi_{i-1/2}) \\ &= \frac{1}{4}(f_{i+2} - 2f_{i+1} + 2f_{i-1} - f_{i-2})(\phi_{i+1} - \phi_{i-1})\end{aligned} \tag{S17}$$

and

$$\delta^3\phi_i\delta f_i = \frac{1}{4}(\phi_{i+2} - 2\phi_{i+1} + 2\phi_{i-1} - \phi_{i-2})(f_{i+1} - f_{i-1}). \tag{S18}$$

Equation (S14) can be easily extended to two dimensions. We call the derived stencil G_1.
However, we can repeat the previous derivation on a different grid, along the two axes $\vec{a}_1 = (1,1)$ and $\vec{a}_2 = (-1,1)$. In this case, the lattice spacing is $h\sqrt{2}$. On this skewed grid,

$$\partial_1 \to \frac{1}{h\sqrt{2}}\delta_1 \tag{S19}$$

where

$$\delta_1 u_{i,j} = u_{i+1/2,j+1/2} - u_{i-1/2,j-1/2}. \tag{S20}$$

This resulting stencil is called G_2.
Then we can average the two derived representations for the derivative, to create a more isotropic stencil G. The weights taken from [1] are

$$G = (2/3) \times G_1 + (1/3) \times G_2. \tag{S21}$$

References

[1] TN Krishnamurti, HS Bedi, and VM Hardiker. An introduction to global spectral modeling. *Mathematical Geology*, 31(8):1011–1012, 1999.
